# Loss of β-catenin in resident cardiac fibroblasts attenuates fibrosis induced by pressure overload in mice

**DOI:** 10.1038/s41467-017-00840-w

**Published:** 2017-09-28

**Authors:** Fu-Li Xiang, Ming Fang, Katherine E. Yutzey

**Affiliations:** The Heart Institute, Division of Molecular Cardiovascular Biology, Cincinnati Children’s Hospital Medical Center, 240 Albert Sabin Way, ML 7020, Cincinnati, OH 45229 USA

## Abstract

Cardiac fibrosis is characterized by excessive extracellular matrix deposition that contributes to compromised cardiac function and potentially heart failure. Cardiac pressure overload resulting from trans-aortic constriction in mice leads to cardiac fibrosis and increased Wnt/β-catenin signaling in cardiac fibroblasts. Here, we conditionally induce β-catenin loss of function in resident cardiac fibroblasts using *Tcf21* ^*MerCreMer*^ or in activated cardiac fibroblasts using periostin (*Postn*)^*MerCreMer*^. We show that β-catenin loss of function in cardiac fibroblasts after trans-aortic constriction significantly preserves cardiac function, and reduces interstitial fibrosis but does not alter the numbers of activated or differentiated cardiac fibroblasts in vivo. However, β-catenin is specifically required in resident cardiac fibroblasts for fibrotic excessive extracellular matrix gene expression and binds *Col3a1* and *Postn* gene sequences in cultured cardiac fibroblasts after induction of Wnt signaling. Moreover, cardiomyocyte hypertrophy is blunted with cardiac fibroblast-specific loss of β-catenin after trans-aortic constriction in vivo. Thus, Wnt/β-catenin signaling in resident cardiac fibroblasts is required for excessive extracellular matrix gene expression and collagen deposition after trans-aortic constriction.

## Abstract (web summary)

Understanding the mechanisms causing cardiac fibrosis is key to prevention and therapy development of many heart diseases. Here, the authors show that Wnt/β-catenin signaling in resident cardiac fibroblasts is required for deposition of fibrotic extracellular matrix and the regulation of cardiomyocyte hypertrophy in a mouse model of heart fibrosis.

## Introduction

Cardiac fibrosis, commonly seen with a variety of cardiac injuries, can significantly reduce tissue compliance and disrupt cardiac conduction, thus contributing to morbidity and mortality associated with heart disease^1–3^. The hallmark of cardiac fibrosis is increased fibrillar collagen, which contributes to reduced cardiac output and can ultimately lead to heart failure^4^. Cardiac fibroblasts (CFs) that arise from epicardial and endothelial progenitors in the developing heart are the predominant collagen-producing cell type in pathologic cardiac fibrosis^5–7^. Although these resident CFs maintain a quiescent phenotype under physiological conditions, they can be activated in response to various types of cardiac injury^2, 7, 8^. Importantly, the regulatory mechanisms that lead to increased collagen production from resident CFs under pathophysiologic conditions, ultimately leading to heart failure, have not been fully elucidated.

Wnt/β-catenin signaling is induced in areas of inflammation, scar formation, and epicardial activation in mouse models of autoimmune endocarditis and ischemic injury^9–11^. However the role of Wnt/β-catenin signaling in myocardial interstitial fibrosis independent from scar formation has not been determined. In addition, the requirement for Wnt/β-catenin signaling specifically in resident CFs and direct downstream targets related to cardiac fibrosis have not been reported previously. Recently developed inducible Cre-expressing lines are effective for manipulation of gene expression in resident CF lineages. A tamoxifen (TAM)-inducible *Tcf21* ^*MerCreMer*^ (*Tcf21* ^*MCM*^) Cre-expressing transgene is highly effective for manipulation of gene expression in adult cardiac resident fibroblasts, which are the primary source of activated fibroblasts in the adult mouse heart^7, 12, 13^. Kanisicak et al.^7^ have developed an inducible knock-in Cre line *periostin (Postn)* ^*MCM*^ that efficiently marks activated myofibroblasts in the fibrotic heart and other organs. These Cre drivers are not expressed in infiltrating immune cells, endothelial cells, or cardiomyocytes, and thus can be used for specific manipulation of resident and activated CFs^7^. Specific regulatory mechanisms that contribute to myofibroblast activation vs. fibroblast differentiation and collagen synthesis are not well-defined, particularly in hypertensive heart failure models^14^. Thus, the use of *Tcf21* ^*MCM*^ for genetic manipulation of resident CFs and *Postn* ^*MCM*^ to specifically target activated CFs is an effective approach for studies of CF-specific regulatory mechanisms in cardiac fibrosis.

The requirements for Wnt/β-catenin signaling specifically in resident and activated CFs after cardiac pressure overload were examined using *Tcf21* ^*MCM*^ ^12^- and *Postn* ^*MCM*^ ^7^-mediated loss of β-catenin (*Ctnnb1* ^*fl/fl*^). Here, we demonstrate that cardiac pressure overload leads to increased Wnt/β-catenin signaling in CFs, and loss of β-catenin in either *Tcf21* ^*MCM*^ or *Postn* ^*MCM*^ lineage results in improved cardiac function, blunted cardiac hypertrophy, reduced interstitial fibrosis and decreased expression of fibrotic extracellular matrix (ECM) protein genes 8 weeks post trans-aortic constriction (TAC). However, β-catenin loss of function (LOF) in CFs directly reduces *Col1a1*, *Col3a1*, and *Postn* gene expression and indirectly blunts cardiomyocyte hypertrophy. Together, these data support a regulatory role for Wnt/β-catenin signaling in fibrotic ECM gene expression and collagen deposition in CFs after TAC-induced pressure overload.

## Results

### Wnt/β-catenin signaling is activated in CFs after TAC

**Fig. 1:**
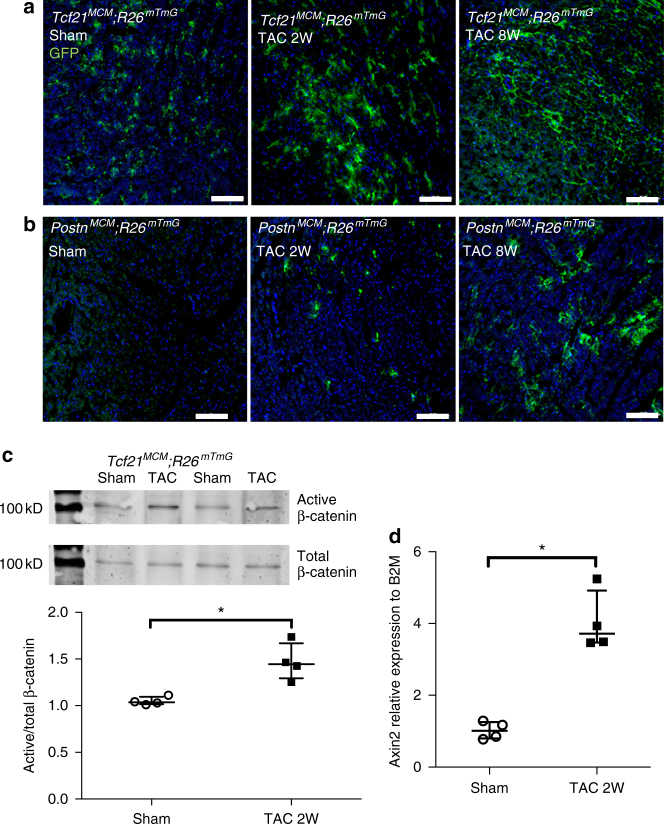
Cardiac fibroblast (CF) lineages express *Tcf21* and *Postn* tamoxifen-inducible Cre, and Wnt/β-catenin is activated after TAC. *Tcf21* ^*MCM*^ *;R26* ^*mTmG*^ and *Postn* ^*MCM*^ *;R26* ^*mTmG*^ mice were subjected to TAC and Cre-activity was induced by TAM food from day 0 after operation. **a** GFP-labeled CFs (*green*) were observed in *Tcf21* ^*MCM*^ *;R26* ^*mTmG*^ sham-operated hearts 2 and 8 weeks after TAC with continuous TAM. **b** GFP-labeled CFs were only observed in *Postn* ^*MCM*^ *;R26* ^*mTmG*^ after TAC but not sham-operated hearts. *Scale bar* = 100 µm. **c** Active and total β-catenin protein expression was determined by western blotting in GFP^+^ CF extracts isolated from *Tcf21* ^*MCM*^ *;R26* ^*mTmG*^ mice 2 weeks after TAC or sham operations. β-catenin signaling activation was determined by the ratio of activated to total β-catenin protein. **d** Expression levels of the Wnt pathway downstream gene *Axin2* in GFP + CFs isolated from *Tcf21* ^*MCM*^ *;R26* ^*mTmG*^ 2 weeks after TAC or sham operations were determined by quantitative PCR relative to B2M. Data points are shown with median and interquartile ranges indicated. Statistical significance was determined using unpaired Mann–Whitney *U* tests: *P < 0.05. *N* = 4 mice per group

During pathologic fibrotic remodeling of the heart, resident Tcf21-expressing CFs are activated and induce expression of periostin (Postn)^7^. TAM-inducible Cre-mediated recombinase activity in Tcf21 and Postn lineages was examined in *Tcf21* ^*MCM*^ ^12^ and *Postn* ^*MCM*^ ^7^ mouse lines bred with the *Rosa26* ^*mTmG*^ (*R26* ^*mTmG*^) green fluorescent protein (GFP)^+^ reporter line^15^. Mice were subjected to trans-aortic constriction (TAC) resulting in pressure overload compared to sham-operated controls (Supplementary Table 1). Adult male (2–3 months of age) *Tcf21* ^*MCM*^ *;R26* ^*mTmG*^ and *Postn* ^*MCM*^ *;R26* ^*mTmG*^ mice were subjected to TAC or sham surgery and were fed TAM to induce Cre activity from day 0 post operation for 2 or 8 weeks. In sham-operated hearts, GFP expression, indicative of Cre-mediated recombination, was detected in *Tcf21* ^*MCM*^ *;R26* ^*mTmG*^ mice (Fig. 1a), but little GFP immunoreactivity was observed in *Postn* ^*MCM*^ *;R26* ^*mTmG*^ mice in the absence of injury (Fig. 1b). Thus, Cre is active in *Tcf21* ^*MCM*^, but not *Postn* ^*MCM*^, lineage CFs under basal conditions. Both *Postn* ^*MCM*^ and *Tcf21* ^*MCM*^ mice exhibit Cre-mediated recombination in CFs at 2 and 8 weeks post TAC (Fig. 1a, b), as evidenced by positive immunostaining of GFP, although *Tcf21* ^*MCM*^ robustly labeled more CFs compared to *Postn* ^*MCM*^. Tcf21 and Postn lineage CFs are present in regions with fibrillar collagen (Col)1/3 deposition after TAC (Supplementary Fig. 1), demonstrating a close relationship between CFs with Cre activity and fibrotic ECM.

The activation of Wnt/β-catenin signaling in CFs during pathologic remodeling was examined in Tcf21 lineage GFP^+^ cells after TAC. β-catenin protein activation is increased in isolated CFs after pressure overload in *Tcf21* ^*MCM*^ *;R26* ^*mTmG*^ hearts, relative to sham-operated controls, as determined by western analysis (Fig. 1c). In addition, gene expression of *Axin2*, a direct downstream target of Wnt/β-catenin signaling, also is increased in isolated *Tcf21* ^*MCM*^ *;R26* ^*mTmG*^ GFP^+^ cells after TAC, relative to sham-operated animals (Fig. 1d). Thus, Wnt/β-catenin signaling is increased in resident Tcf21 lineage CFs after pressure overload resulting from TAC.

### Cardiac function is improved with loss of β-catenin in CFs

The requirements for β-catenin in resident and activated CFs during cardiac fibrotic remodeling were determined using mice with a floxed β-catenin allele (*Ctnnb1* ^*fl/fl*^) (Supplementary Fig. 2A) that results in β-catenin LOF in the presence of active Cre^16^. These mice were bred with *Tcf21* ^*MCM*^ and *Postn* ^*MCM*^ Cre-driver lines for TAM-responsive β-catenin LOF in resident fibroblasts and activated myofibroblasts, respectively. Analysis of β-catenin LOF in CFs was performed in *Tcf21* ^*MCM*^ *;R26* ^*mTmG*^ *;Ctnnb1* ^*fl/fl*^ mice and Cre-negative littermate controls subjected to TAC surgery and maintained on TAM (Supplementary Fig. 2C). Loss of β-catenin protein was confirmed in GFP^+^ cells isolated from *Tcf21* ^*MCM*^ *;R26* ^*mTmG*^;*Ctnnb1* ^*fl/fl*^ hearts 4 weeks after TAC (Supplementary Fig. 2D). Wnt/β-catenin signaling also was reduced as indicated by lack of *Axin2* gene induction in *Tcf21* ^*MCM*^ *;R26* ^*mTmG*^ *;Ctnnb1* ^*fl/fl*^ β-catenin LOF sorted GFP^+^ cells relative to *Tcf21* ^*MCM*^ *;R26* ^*mTmG*^ control GFP^+^ cells after TAC (Supplementary Fig. 2E). Thus, β-catenin protein expression and TAC-induced Wnt signaling activation, as indicated by *Axin2* gene induction, are reduced in *Tcf21* ^*MCM*^ *;Ctnnb1* ^*fl/fl*^ CFs relative to Cre-negative controls.

**Fig. 2:**
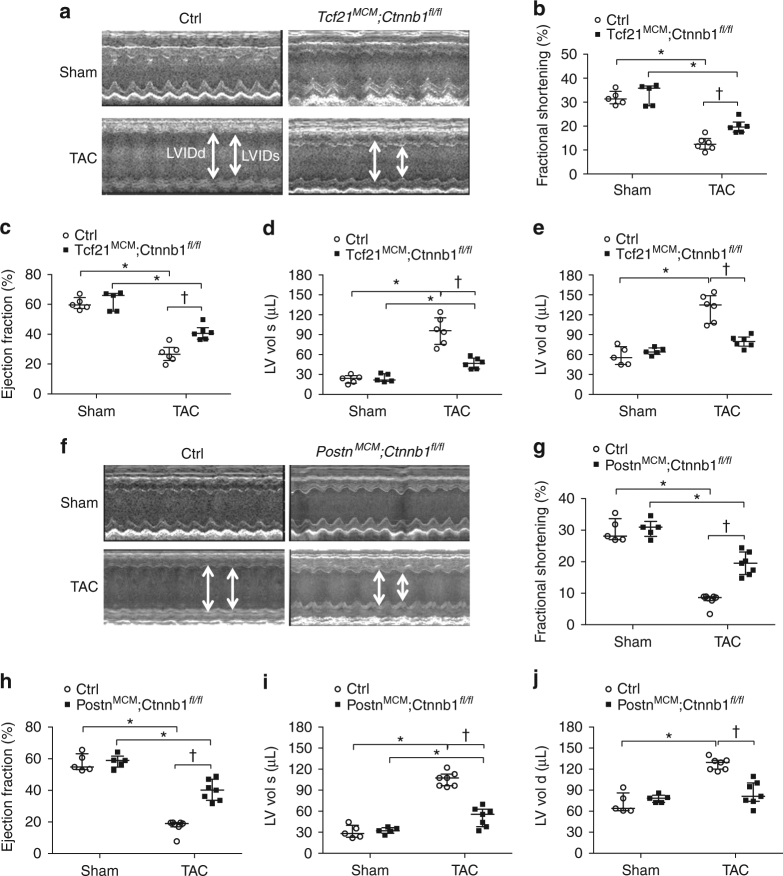
Loss of β-catenin in Tcf21 or Postn CF lineages leads to improved cardiac function 8 weeks post TAC. *Tcf21* ^*MCM*^ *;Ctnnb1* ^*fl/fl*^ and *Postn* ^*MCM*^ *;Ctnnb1* ^*fl/fl*^ mice with corresponding Cre-negative controls were subjected to sham or TAC operations. Cre-activity was induced by TAM food for 8 weeks from day 0 after operation, and cardiac function was measured by echocardiography. Representative M-mode tracing for *Tcf21* ^*MCM*^ *;Ctnnb1* ^*fl/fl*^ (**a**) and *Postn* ^*MCM*^ *;Ctnnb1* ^*fl/fl*^ (**f**) with corresponding Cre-negative controls at 8 weeks post sham or TAC operations are shown. *Arrows* indicate left ventricular internal diameter end diastole (LVIDd, *left*) and end systole (LVIDs, *right*). **b**, **g** Fractional shortening (FS), (**c**, **h**) ejection fraction (EF), (**d**, **i**) LV systolic (LV Vol s), and (**e**, **j**) LV diastolic volume (LV Vol d) were quantified. No differences were observed between sham groups. TAC significantly impaired cardiac function compared to sham control after 8 weeks. Compared to Cre-negative littermate controls, FS, EF, and LV Vol were significantly improved in both *Tcf21* ^*MCM*^ *;Ctnnb1* ^*fl/fl*^ (**b**–**e**) and *Postn* ^*MCM*^ *;Ctnnb1* ^*fl/fl*^ (**g**–**j**) mice at 8 weeks post TAC. Data points are shown with median and interquartile ranges indicated. Statistical significance was determined by Kruskal–Wallis tests followed Mann–Whitney *U* tests for pairwise comparisons using Bonferonni adjustments to control for multiple testing. *P < 0.05 vs. Sham, ^†^ *P* < 0.05 vs. Control TAC. *N* = 5–7 mice per group

Adult male *Tcf21* ^*MCM*^ *;Ctnnb1* ^*fl/fl*^ *, Postn* ^*MCM*^ *;Ctnnb1* ^*fl/fl*^ and Cre-negative littermate controls were subjected to either TAC or sham surgery, followed by feeding with TAM to induce Cre activity beginning at day 0 post operation and continuing for 8 weeks (Supplementary Fig. 2B). At 8 weeks post TAC, M-mode echocardiography was performed to examine cardiac function and dimensions (Fig. 2a, f). Cardiac dysfunction, as indicated by reduced fractional shortening (FS) and ejection fraction (EF), was observed in mice subjected to TAC after 8 weeks compared to sham groups (Supplementary Table 1). However, both *Tcf21* ^*MCM*^ *;Ctnnb1* ^*fl/fl*^ and *Postn* ^*MCM*^ *;Ctnnb1* ^*fl/fl*^ mice demonstrated significantly preserved cardiac function as shown by higher FS (Fig. 2b, g), improved EF (Fig. 2c, h), and less left ventricular (LV) dilation (Fig. 2d, i) compared to Cre-negative controls. Additional *Tcf21* ^*MCM*^ and *Postn* ^*MCM*^ control mice were evaluated for cardiac function after 8 weeks of TAM administration, and no differences were observed relative to Cre-negative controls at baseline (Supplementary Table 2). Moreover, no differences in mortality were observed 8 weeks after surgery among the groups. Thus, loss of β-catenin in *Tcf21* ^*MCM*^ or *Postn* ^*MCM*^ lineages leads to preserved cardiac function, relative to Cre-negative controls, 8 weeks after TAC-induced pressure-overload injury.

### CF-specific loss of β-catenin attenuates cardiac hypertrophy

**Fig. 3:**
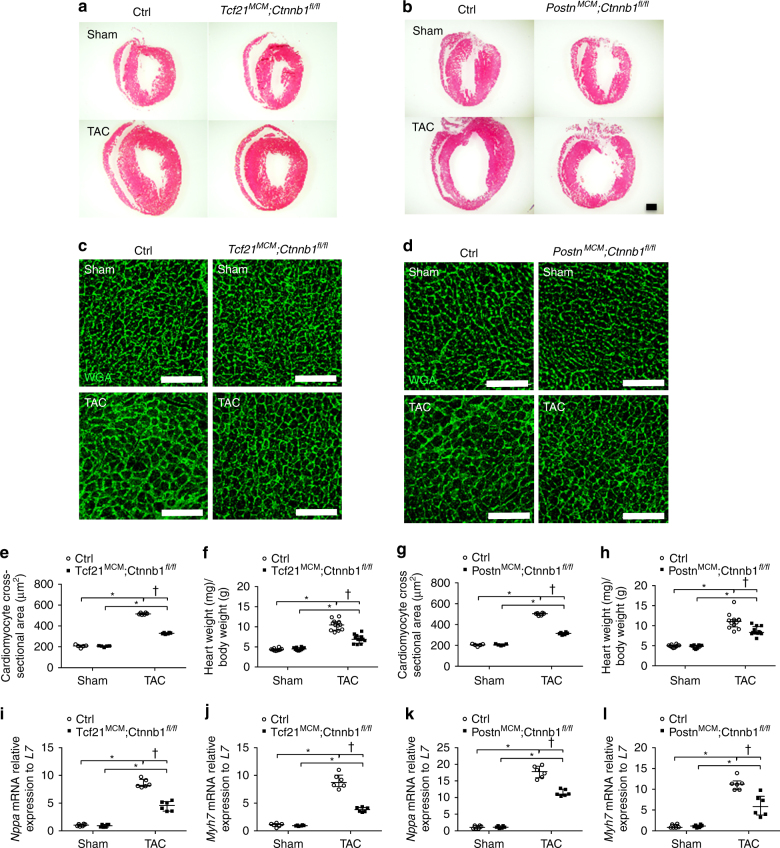
Loss of β-catenin in Tcf21 or Postn lineage attenuates cardiac hypertrophy 8 weeks post TAC. Representative images of hematoxylin and eosin (**a**, **b**, *scale bar* = 1mm) and immunofluorescence staining of WGA (*green*, **c**, **d**, *scale ba*r = 100 μm) from *Tcf21* ^*MCM*^ *;Ctnnb1* ^*fl/fl*^ (**a**, **c**) and *Postn* ^*MCM*^ *;Ctnnb1* ^*fl/fl*^ (**b**, **d**) are shown. Compared to Cre-negative controls, loss of β-catenin in Tcf21 (**e**, **f**) or Postn (**g**, **h**) lineages significantly decreased cardiac hypertrophy 8 weeks post TAC as determined by cardiomyocyte cross-section area (**e**) and (**g**) and heart-to-body weight ratio (**f**) and (**h**). **i**–**l** Gene expression levels of hypertrophic markers *Nppa* and *Myh7* was determined by quantitative PCR of RNA isolated from LV free wall of *Tcf21* ^*MCM*^ *;Ctnnb1* ^*fl/fl*^ (**i**, **j**) and *Postn* ^*MCM*^ *;Ctnnb1* ^*fl/fl*^ (**k**, **l**) at 8 weeks post operation. Data points are shown with median and interquartile ranges indicated. Statistical significance was determined by Kruskal–Wallis tests, followed by Mann–Whitney *U* tests for pairwise comparisons using Bonferonni adjustments to control for multiple testing. *P < 0.05 vs. Sham, ^†^ *P* < 0.05 vs. Control TAC. *N* = 5–12 mice per group

Cardiac pressure overload resulting from TAC leads to a robust cardiac hypertrophy response in addition to induction of increased ECM production and fibrotic remodeling. The interdependence of cardiac fibrosis and myocyte hypertrophy was examined in sham-operated and TAC hearts from *Tcf21* ^*MCM*^ *;Ctnnb1* ^*fl/fl*^ and *Postn* ^*MCM*^ *;Ctnnb1* ^*fl/fl*^ mice and their corresponding Cre-negative littermate controls 8 weeks after TAC or sham surgeries. As expected, control hearts subjected to TAC exhibited increased heart size, indicative of cardiac hypertrophy, relative to sham-operated controls (Fig. 3a, b). However, hearts from *Tcf21* ^*MCM*^ *;Ctnnb1* ^*fl/fl*^ and *Postn* ^*MCM*^ *;Ctnnb1* ^*fl/fl*^ mice were smaller in size compared to Cre-negative littermate controls at 8 weeks post TAC. The extent of cardiomyocyte hypertrophy was determined by individual cardiomyocyte cross-sectional area and also by heart weight-to-body weight ratios at the whole-organ level. In heart cross-sections, cell membranes were visualized by wheat germ agglutinin (WGA) staining (Fig. 3c, d) and cross-sectional areas of cardiomyocytes were quantified. Compared to Cre-negative controls, cardiomyocyte cross-sectional areas were significantly reduced in *Tcf21* ^*MCM*^ *;Ctnnb1* ^*fl/fl*^ and *Postn* ^*MCM*^ *;Ctnnb1* ^*fl/fl*^ mice 8 weeks after TAC (Fig. 3e, g). Likewise, cardiac hypertrophy, as indicated by heart weight-to-body weight ratio (Fig. 3f, h), also was blunted with loss of β-catenin in either Tcf21 or Postn lineage cells. Moreover, messenger RNA (mRNA) expression of cardiac hypertrophy markers *Nppa* and *Myh7* was significantly lower in LV free walls (LVFWs) in *Tcf21* ^*MCM*^ *;Ctnnb1* ^*fl/fl*^ and *Postn* ^*MCM*^ *;Ctnnb1* ^*fl/fl*^ mice compared to controls after TAC (Fig. 3i−l). In addition, no difference in capillary density was observed in Ctrl myocardium compared to *Tcf21* ^*MCM*^ *;Ctnnb1* ^*fl/fl*^ and *Postn* ^*MCM*^ *;Ctnnb1* ^*fl/fl*^ mice 8 weeks after TAC (Supplementary Fig. 3). Together, these results demonstrate that loss of β-catenin in either Tcf21 or Postn lineage CFs leads to decreased cardiac hypertrophy after TAC-induced pressure-overload injury. The reduction in cardiac hypertrophy is likely an indirect effect of a reduced fibrotic response in CFs, since neither of these Cres is expressed in cardiac muscle cells^7^.

### CF-specific loss of β-catenin attenuates fibrosis after TAC

**Fig. 4:**
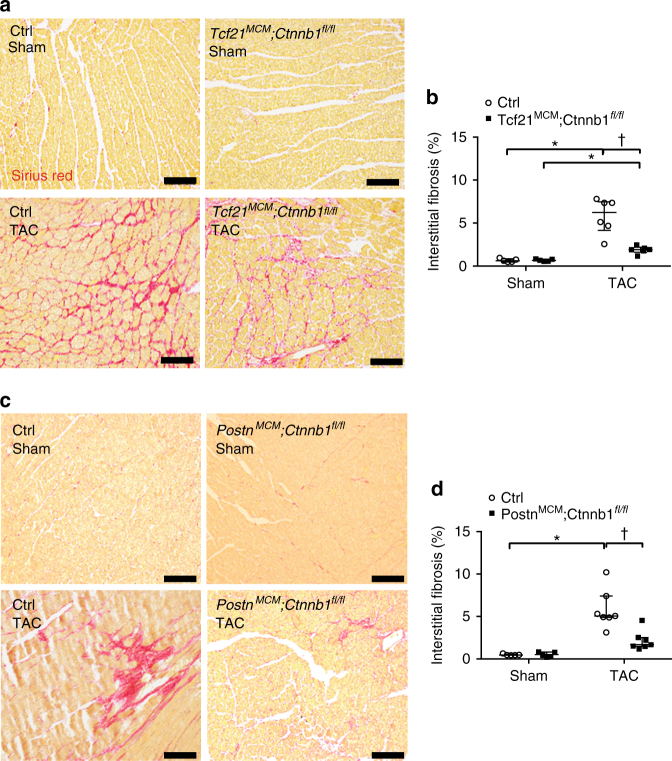
Loss of β-catenin in Tcf21 or Postn lineages leads to reduced interstitial cardiac fibrosis 8 weeks after TAC. **a**, **c** Heart sections were stained with Sirius Red to visualize fibrosis (*red*). Representative images of interstitial fibrosis are shown for *Tcf21* ^*MCM*^ *;Ctnnb1* ^*fl/fl*^ (**a**) and *Postn* ^*MCM*^ *;Ctnnb1* ^*fl/fl*^ (**c**) with corresponding Cre-negative controls 8 weeks after sham or TAC operations. *Scale bar* = 100 μm. **b**, **d** The percent total area of interstitial fibrosis as indicated by Sirius Red staining was quantified. Data points are shown with median and interquartile ranges indicated. Statistical significance was determined by Kruskal–Wallis tests followed Mann–Whitney *U* tests for pairwise comparisons using Bonferonni adjustments to control for multiple testing. *P < 0.05 vs. Sham, ^†^ *P* < 0.05 vs. Control TAC. *N* = 5–7 mice per group

In order to evaluate the effects of loss of β-catenin on cardiac fibrotic remodeling, collagen deposition in heart sections was visualized by Sirius Red staining (Fig. 4a, c). TAC-induced pressure overload for 8 weeks resulted in robust cardiac fibrosis (Fig. 4b, d) compared to sham-operated control groups. However, loss of β-catenin leads to decreased interstitial fibrosis in both *Tcf21* ^*MCM*^ *;Ctnnb1* ^*fl/fl*^ and *Postn* ^*MCM*^ *;Ctnnb1* ^*fl/fl*^ mice, relative to Cre-negative controls at 8 weeks post TAC (Fig. 4b, d). Thus, loss of β-catenin in either activated myofibroblasts or resident CFs leads to reduced collagen deposition during cardiac fibrotic remodeling.

**Fig. 5:**
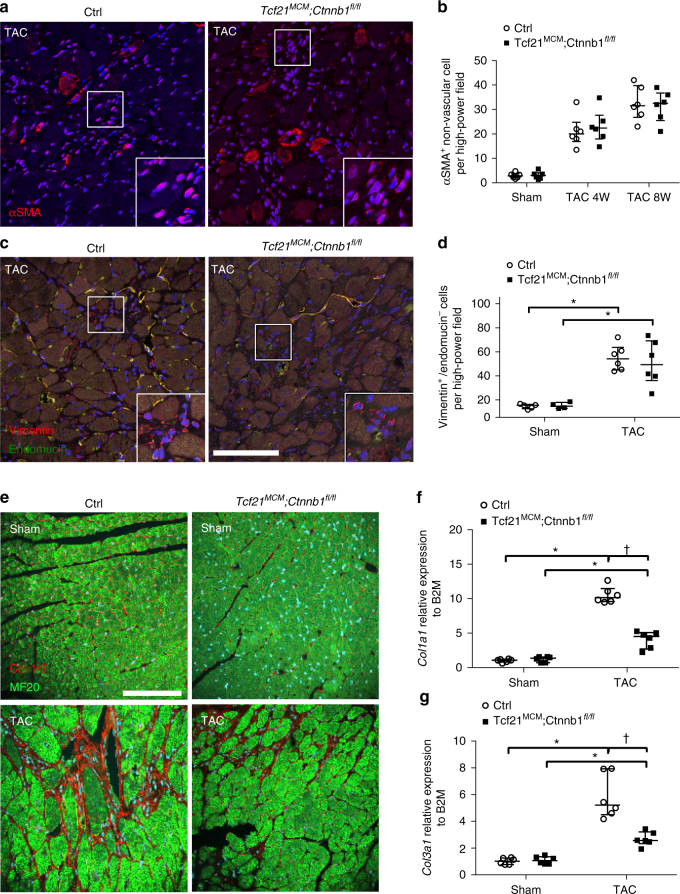
Loss of β-catenin in Tcf21 lineage cells does not affect CF activation or total CF numbers but does lead to decreased *Col1a1* and *Col3a1* expression 8 weeks post TAC. **a** Representative images of αSMA staining (*red*, in *insets*) in *Tcf21* ^*MCM*^ *;Ctnnb1* ^*fl/fl*^ and Cre-negative control hearts 8 weeks after TAC are shown. **b** Non-vascular αSMA^+^ cells (αSMA^+^ cells surrounding vessel lumens were excluded from the analysis) were identified as activated CFs and quantified. **c** Representative images of vimentin (*red*) and endomucin (*green*) co-staining in *Tcf21* ^*MCM*^ *;Ctnnb1* ^*fl/fl*^ and Cre-negative control hearts 8 weeks after TAC are shown. Vimentin^+^/endomucin^−^ cells were identified as CFs (*red* cells in insets) and **d** quantified. **e** Representative images for collagen 1/3 (Col1/3) immunofluorescence (*red*) co-stained with MF20 (*green*) in *Tcf21* ^*MCM*^ *;Ctnnb1* ^*fl/fl*^ and Cre-negative control hearts are shown. *Scale bar* = 100 μm. Quantitative PCR analysis of *Col1a1* (**f**) and *Col3a1* (**g**) was performed using mRNA isolated from LV free wall at 8 weeks post operation. Data points are shown with median and interquartile ranges indicated. Statistical significance was determined by Kruskal–Wallis tests followed Mann–Whitney *U* tests for pairwise comparisons using Bonferonni adjustments to control for multiple testing. *P < 0.05 vs. sham, ^†^ *P* < 0.05 vs. Control TAC. *N* = 4–8 mice per group

Development of cardiac fibrosis under pathological conditions includes CF activation and proliferation followed by increased ECM production^8, 17^. Since similarly reduced cardiac function and fibrosis were observed in *Tcf21* ^*MCM*^ *;Ctnnb1* ^*fl/fl*^ and *Postn* ^*MCM*^ *;Ctnnb1* ^*fl/fl*^ mice after TAC, *Tcf21* ^*MCM*^ *;Ctnnb1* ^*fl/fl*^ mice with β-catenin LOF in both resident CF and activated myofibroblast lineages^7^ were used in these analyses. CF activation, proliferation, and total cell numbers were determined in *Tcf21* ^*MCM*^ *;Ctnnb1* ^*fl/fl*^ mice and Cre-negative littermate controls 4 weeks and 8 weeks after TAC. CF activation was evaluated by quantification of the number of individual αSMA^+^ cells exclusive of vascular smooth muscle. No difference in the number of αSMA^+^ cells was observed between *Tcf21* ^*MCM*^;*Ctnnb1* ^*fl/fl*^ mice and Cre-negative littermate controls in sham-operated, 4-week TAC or 8-week TAC animals (Fig. 5a, b). Thus, β-catenin LOF does not alter the number of activated myofibroblasts as indicated by αSMA after pressure overload.

**Fig. 6:**
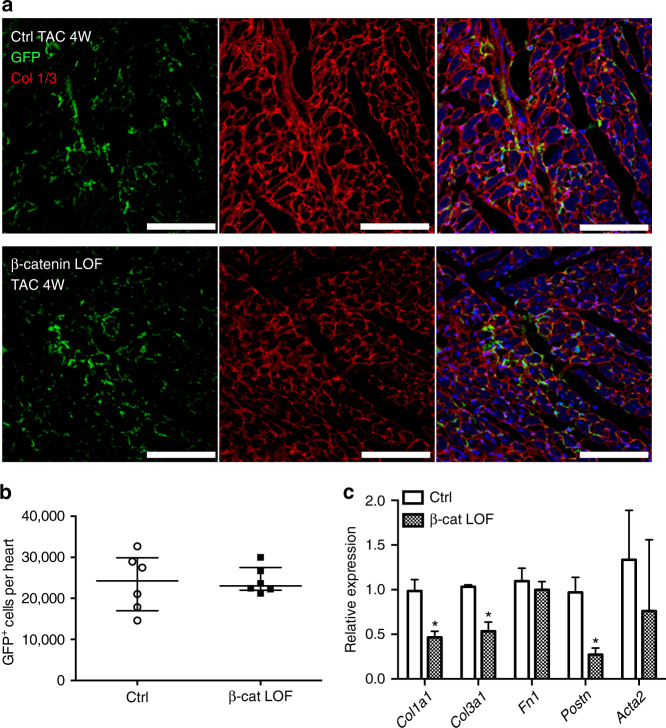
The total number of Tcf21GFP^+^ CFs is not altered, but fibrotic ECM gene expression is reduced, with loss of β-catenin in Tcf21 lineage CFs 4 weeks after TAC. **a** Representative images of *Tcf21* ^*MCM*^ *;R26* ^*mTmG*^ *;Ctnnb1* ^*fl/+*^ (Ctrl, *top*) and *Tcf21* ^*MCM*^ *;R26* ^*mTmG*^ *;Ctnnb1* ^*fl/fl*^ (β-catenin LOF, *bottom*) with immunostaining for GFP (*green*) and collagen 1/3 (Col1/3, *red*) are shown. **b** GFP^+^ Tcf21 lineage CFs were isolated by FACS (fluorescence-actived cell sorting) and the number of GFP^+^ cells was quantified in *Tcf21* ^*MCM*^ *;R26* ^*mTmG*^ *;Ctnnb1* ^*fl/fl*^ and control mice 4 weeks after TAC. **c** mRNA was isolated from sorted Tcf21 lineage GFP^+^ cells. Expression of *Col1a1*, *Col3a1*, and *Postn* was significantly lower in CFs from *Tcf21* ^*MCM*^ *;R26* ^*mTmG*^ *;Ctnnb1* ^*fl/fl*^ mice compared to controls, but *Fn1* and *Acta2* was not changed as determined by quantitative PCR. Data points are shown with median and interquartile ranges indicated. Statistical significance was determined using unpaired Mann–Whitney *U* tests: *P < 0.05. *N* = 6 mice per groupFluorescence-actived cell sorting (FACS)

CFs can be marked by expression of the intermediate filament vimentin^18^ exclusive of vimentin + endothelial cells that also express the endothelial marker endomucin^19^. Thus, the overall number of CFs was quantified based on the vimentin^+^/endomucin^−^ cell population (Fig. 5c). Quantification of vimentin^+^/endomucin^−^ cells showed no difference in the CF population between *Tcf21* ^*MCM*^ *;Ctnnb1* ^*fl/fl*^ mice and Cre-negative littermate controls 8 weeks after sham or TAC surgeries (Fig. 5d). Thus, loss of β-catenin in the Tcf21 CF lineage does not affect the total number of vimentin^+^/endomucin^−^ CFs 8 weeks after TAC. In addition, total CF cell numbers based on the GFP reporter expression were determined after cell sorting of *Tcf21* ^*MCM*^ *;R26* ^*mTmG*^ *;Ctnnb1* ^*fl/fl*^ and *Tcf21* ^*MCM*^ *;R26* ^*mTmG*^ control GFP + cells 4 weeks after TAC. Notably, there was no significant difference in the total number of GFP + cells isolated in β-catenin LOF relative to controls apparent by histology (Fig. 6a) or flow cytometry of sorted GFP^+^ cells (Fig. 6b). Additionally, no changes in CF proliferation, as indicated by phospho-histone 3 (pHH3) and GFP costaining (Supplementary Fig. 4A), or in cell death, as indicated by TUNEL and GFP costaining, were detected in mice with Tcf21-mediated loss of β-catenin after TAC, relative to controls (Supplementary Fig. 4B). Thus, loss of β-catenin in the Tcf21 lineage does not lead to reduced CF activation or total CF cell numbers in hearts with decreased fibrosis after TAC.

The effects of Wnt/β-catenin signaling in regulating CF activation, ECM production, and proliferation under pathological conditions were further investigated in cultured adult mouse (2-month) CFs stimulated with angiotensin (Ang) II. Treatment with Ang II (100 nM) for 48 h significantly induced CF activation, as determined by αSMA staining (Supplementary Fig. 5A and B) and *Postn* gene expression, as well as increased fibrotic ECM mRNA expression (Supplementary Fig. 5C). Interestingly, Ang II treatment also promotes Wnt/β-catenin signaling, as indicated by increased *Axin2* expression (Supplementary Fig. 5C), and inhibition of Wnt/β-catenin signaling with XAV939 abrogates CF activation and ECM production in response to Ang II (Supplementary Fig. 5A−C). However, CF proliferation was not affected by Ang II or by Wnt/β-catenin signaling inhibition (Supplementary Fig. 5D−F). These data support a requirement for Wnt/β-catenin signaling in CF activation and ECM expression, but not proliferation, in response to an AngII-mediated fibrotic stimulus.

### Collagen expression is reduced with loss of β-catenin in CFs

Since no change in myofibroblast activation or overall numbers of CFs was observed, regulation of fibrillar collagen protein and gene expression was examined as a direct consequence of β-catenin LOF in CFs. By immunofluorescence, Col1/3 (Fig. 5e) protein expression is reduced in the myocardium of *Tcf21* ^*MCM*^ *;Ctnnb1* ^*fl/fl*^ mice, compared to controls, 8 weeks after TAC. Moreover, *Col1a1* and *Col3a1* mRNA expression also is reduced in LVFW of *Tcf21* ^*MCM*^ *;Ctnnb1* ^*fl/fl*^ mice compared to controls (Fig. 5f, g). Fibrotic ECM gene expression was examined specifically in GFP + CF isolated from *Tcf21* ^*MCM*^ *;R26* ^*mTmG*^ *;Ctnnb1* ^*fl/fl*^ β-catenin LOF hearts compared to *Tcf21* ^*MCM*^ *;R26* ^*mTmG*^ control hearts 4 weeks after TAC (Fig. 6c). mRNA expression of *Cola1*, *Col3a1*, and *Postn* was significantly decreased in CFs with β-catenin LOF, while no difference in *Acta2* (αSMA) expression was observed. Collectively, these results demonstrate that loss of β-catenin in Tcf21 lineage CFs after TAC specifically leads to reduced collagen mRNA and protein expression in response to pressure-overload injury.

### Wnt/β-catenin is activated by Tgfβ and promotes ECM gene expression

**Fig. 7:**
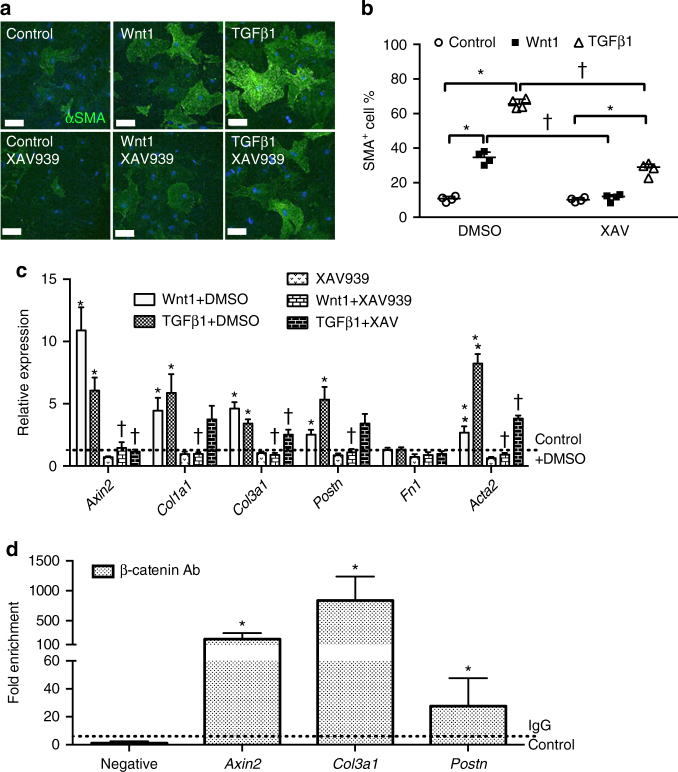
Wnt/β-catenin signaling induces cultured CF activation and ECM gene expression downstream of Tgfβ signaling in vitro. **a** Representative images of αSMA (*green*) staining in cultured P60 CFs under indicated conditions are shown. *Scale bar* = 100 μm. **b** Wnt1 (100 ng/ml) and TGFβ1 (10 ng/ml) induces significantly more αSMA^+^ CFs relative to DMSO controls in cultured P60 CFs. Inhibition of Wnt/β-catenin signaling by XAV939 (5 μM) abrogated Wnt1-induced αSMA expression in CF and also partially suppressed TGFβ1-induced αSMA expression. *P < 0.05 vs. DMSO groups, ^†^ *P* < 0.05 vs. Control. **c** mRNA expression of the Wnt target gene *Axin2*, and ECM genes *Col1a1*, *Col3a1*, *Postn*, and *Acta2* in P60 CFs with indicated treatments was measured by quantitative PCR. *P < 0.05 vs. control, ^†^ *P* < 0.05 vs. DMSO. *N* = 4 independent cultures per group. Statistical significance was determined by Kruskal–Wallis tests followed by Mann–Whitney *U* tests for pairwise comparisons using Bonferonni adjustments to control for multiple testing. **d** ChIP assays were performed using P0 cultured CFs treated with BIO (2 μM) to determine the binding of β-catenin in protein complexes with TCF/LEF consensus containing proximal sequences of *Axin2*, *Col3a1*, and *Postn* genes. Compared to IgG negative control, binding of β-catenin to *Col3a1* and *Postn* was significantly enriched. *Axin2* was used as positive control. *N* = 4 independent cultures per group. Fold enrichment of DNA binding in ChIP assays was analyzed using one sample Wilcoxon signed-rank test compared to one. Data points are shown with median and interquartile ranges indicated. *P < 0.05 vs. IgG control

The necessity and sufficiency of Wnt/β-catenin signaling in regulating CF activation and ECM gene expression was further investigated in cultured adult mouse (2-month) CFs. Treatment with Wnt1 (100 ng/ml) for 48 h significantly induced CF activation as determined by αSMA staining (Fig. 7a, b) and *Acta2* mRNA expression (Fig. 7c). This increase could be blocked by treatment with the Wnt/β-catenin pathway inhibitor XAV939. Likewise, TGFβ1 treatment leads to β-catenin activation (Supplementary Fig. 6) and increased *Axin2* mRNA expression (Fig. 7c) indicative of activated Wnt/β-catenin signaling downstream of TGFβ signaling. Interestingly, XAV939 (5 μM) treatment also attenuated TGFβ1-induced CF activation (Fig. 7b, c), providing additional evidence that Wnt/β-catenin signaling acts downstream of TGFβ in regulating CF activation. Thus, Wnt/β-catenin signaling is sufficient to promote CF activation in cell culture and also contributes to TGFβ-mediated CF activation.

The direct effects of Wnt/β-catenin signaling on CF differentiation and ECM gene expression were examined. Wnt1 treatment, like TGFβ1 treatment, leads to increased expression of *Col1a1*, *Col3a1*, and *Postn*, which was attenuated by XAV939 (Fig. 7c). In order to determine if a β-catenin/LEF/TCF transcriptional complex directly binds to ECM genes in CFs, chromatin immunoprecipitation (ChIP) assays were performed using an anti-β-catenin antibody. P0 CFs were treated with the Wnt/β-catenin signaling activator BIO (2 µM) for 48 h before ChIP. Primers amplifying LEF/TCF binding consensus sequences in *Axin2* intron 1 were used as positive control for β-catenin/LEF/TCF binding^20^. Significant binding enrichment of β-catenin also was found in *Col3a1* and *Postn* gene sequences that contain TCF/LEF consensus sequences (Fig. 7d and Supplementary Fig. 7), supporting a direct regulatory interaction. Thus, Wnt/β-catenin signaling is sufficient to promote ECM gene expression, and β-catenin occupies *Col3a*1 and *Postn* genomic sequences containing TCF/LEF consensus sequences in CFs after Wnt/β-catenin pathway induction. Together with in vivo β-catenin LOF studies, these data support a direct regulatory interaction by which collagen gene expression is induced by Wnt/β-catenin signaling during cardiac fibrotic remodeling.

## Discussion

In response to cardiac pressure overload injury, β-catenin protein is activated and *Axin2* gene expression is induced in CFs, indicative of increased Wnt/β-catenin signaling in cardiac fibrosis. Loss of β-catenin in either *Tcf21* ^*MCM*^ or *Postn* ^*MCM*^ lineage after TAC significantly improves cardiac function and attenuates cardiac fibrosis after TAC-induced pressure overload injury in mice. Interestingly, reduction of cardiac fibrosis due to CF-specific loss of β-catenin leads to decreased cardiomyocyte hypertrophy, despite the continued pressure overload stimulus. Loss of β-catenin in the *Tcf21* ^*MCM*^ lineage after TAC does not lead to attenuated activation of CFs as indicated by αSMA^+^, or reduction in the total number of CFs, as indicated by vimentin^+^/endomucin^−^ immunostaining or expression of the Tcf21 GFP reporter. However, fibrillar collagen protein and mRNA expression is reduced with loss of β-catenin in CFs after TAC. In cultured CFs, Wnt1 treatment induces CF activation and collagen gene expression. Wnt pathway activation also contributes to TGFβ1-induced CF activation and ECM gene induction. In addition, β-catenin occupancy of *Col3a1* and *Postn* gene sequences containing TCF/LEF binding sequences was demonstrated by ChIP. Together, these data support an important role for Wnt/β-catenin signaling in directly promoting ECM gene expression in CFs and fibrotic remodeling after TAC-induced hypertensive injury in mice (Supplementary Fig. 8).

Here, we demonstrate a requirement for Wnt/β-catenin signaling specifically in resident CFs in cardiac fibrosis after TAC. Previously, Wnt/β-catenin signaling has been reported to contribute to cardiac fibrosis in mice after ischemic injury in adults, cryoinjury in neonates, or in autoimmune endocarditis^10, 11, 21^. After ischemia or cryoinjury, Wnt/β-catenin signaling is activated in the epicardium and is required for epicardial-to-mesenchymal activation and expansion of the epicardial-derived cells (EPDCs) after injury^10, 21^. In the adult, these induced EPDCs remain on the surface of the heart and express angiogenic factors after cardiac injury^6, 22, 23^. During cardiac fibrosis resulting from autoimmune myocarditis, Wnt1 and Wnt5a expression is induced in regions of immune cell infiltration, and inhibition of Wnt signaling via administration of soluble pathway inhibitors prevents cardiac fibrosis and improves cardiac function^11^. In contrast, β-catenin loss of function in collagen-expressing cells followed by ischemic injury inhibits scar formation and cardiac remodeling with adverse effects on cardiac function^10^, supporting a role for Wnt/β-catenin signaling in initial scar formation during the acute injury phase, as has also been demonstrated for periostin^24^. Here, we demonstrate that β-catenin is specifically required in resident CFs of Tcf21 and Postn lineages for fibrillar collagen deposition and gene expression after TAC-induced pressure overload. Thus, it is likely that Wnt/β-catenin signaling is activated in multiple cardiac resident and infiltrating cell types depending on the specific type of cardiac injury or stage of fibrotic remodeling^25^.

Multiple signaling pathways, notably Ang II and Tgfβ, have been implicated in aspects of CF activation, myofibroblast differentiation, and collagen remodeling during the cardiac fibrotic response^2^. Here, we demonstrate that Wnt/β-catenin signaling is activated by Ang II or Tgfβ in CFs and is required for CF activation and fibrotic gene expression. Ang II induction of cardiac fibrosis can be mediated by Tgfβ signaling^8^, and our data show that the Wnt/β-catenin pathway is activated by both pathways. Wnt/β-catenin signaling also is required in TGFβ-mediated skin, lung, and liver fibrosis^26–28^. In mouse dermal fibroblasts and human lung fibroblasts, TGFβ signaling induces β-catenin nuclear localization via non-canonical intermediates, including TAK1^26^. This is consistent with our observation that TGFβ induces β-catenin protein stabilization and activation in cultured CFs. Together, these studies support a role for Wnt/β-catenin downstream of Ang II and TGFβ signaling in regulation of CF activation and collagen gene expression in CFs.

Regulation of ECM gene expression directly by Wnt/β-catenin has been reported in fibroblasts from different organs, notably skin^29^. Wnt1 treatment also indirectly induces *Col1a2* promoter activity in dermal fibroblasts^26^. Stabilized β-catenin in dermal fibroblasts is sufficient to increase *Col1a1* expression and promote skin fibrosis in mice^27, 29^. Additionally, loss of β-catenin suppresses bleomycin-induced dermal fibrosis with reduced collagen deposition^27^. Our data show that β-catenin signaling specifically affects fibrillar collagen and periostin expression in CFs. Periostin has a critical role in collagen fibril formation and remodeling that likely contributes to the lack of fibrotic ECM deposition with β-catenin LOF^24, 30^. The specific reduction of fibrotic ECM expression with reduced Wnt/β-catenin signaling in resident or activated CFs suggests that this pathway could be a therapeutic target in the reduction of fibrotic progression in heart failure.

In the current study, we demonstrate that inhibition of fibrotic ECM deposition with loss of β-catenin in resident CFs leads to reduced cardiac hypertrophy and improved cardiac function after TAC. Similarly, CF-specific loss of the transcription factors KLF5 using a transgenic *PostnCre* also results in decreased cardiomyocyte hypertrophy after pressure overload through regulation of *Igf1* expression^31^. In addition, indicators of cardiac fibrotic remodeling are detectable prior to myocardial hypertrophy or dysfunction in mice or human patients with sarcomeric protein mutations that cause familial hypertrophic cardiomyopathy^32, 33^. Taken together, these studies provide evidence for crosstalk between CFs and myocytes very early in the progression of cardiac dysfunction and failure. Multiple signaling pathways including Tgfβ, FGF (fibroblast growth factor), and CTGF (connective tissue growth factor) have been implicated in this crosstalk, but specific signaling and recipient cell types have not been clearly defined^34^. In the current study, cardiomyocyte hypertrophy is attenuated in hearts with reduced fibrotic ECM due to loss of β-catenin specifically in resident CFs. Thus, targeting fibrotic remodeling mediated by resident CFs is important for the development of effective therapeutic approaches for heart failure.

## Methods

### Transgenic and mutant mice

All animal experiments were conducted in accordance with protocols approved by the Institutional Animal Care and Use Committee at Cincinnati Children’s Research Foundation.

*Tcf21* ^*MCM*^ (*Tcf21* ^*MCM*^) mice have been described previously by Acharya et al.^12^ *Postn* ^*MCM*^ (*Postn* ^*MCM*^) knock-in mice were generated in Dr Jeffery Molkentin’s laboratory at Cincinnati Children’s Hospital Medical Center^7^. Mice with *loxp* sites inserted into the β-catenin gene (*Ctnnb1* ^*fl/fl*^) were used for β-catenin loss-of-function studies^15, 16^. *ROSA26* ^*mTmG*^ (*R26* ^*mTmG*^) reporter (Jackson Laboratory, #007576) mice were purchased from Jackson Laboratory. Genotyping was performed using genomic DNA extracted from clipped tails with primers listed in Supplementary Table 3. Unless otherwise indicated, mixed cohorts of male and female mice in a C57BL/6 background were used. Mice were sacrificed by CO_2_ inhalation followed by cervical dislocation at the specified time point for each experiment.

### TAC surgery and TAM-mediated Cre induction

TAC was performed to create pressure overload in a mouse heart^35^. Briefly, male mice (8–10 weeks old, 21–24 g body weight) were anesthetized with 2% isoflurane inhalation. The animals were then placed in a supine position, and orally intubated with 20-gauge tubing and ventilated (Harvard Apparatus Rodent Ventilator, model 845) at 120 breaths per minute (0.1-ml tidal volume). The chest cavity was exposed by cutting open the proximal portion of the sternum. After the aortic arch between the innominate and left common carotid arteries was isolated, the transverse aortic arch was ligated (7–0 Prolene) with an overlying 27-gauge needle, and then the needle was removed, leaving a discrete region of stenosis. Sham-operated mice underwent the identical surgical procedure, including isolation of the aorta, but without placement of the suture. Mice were fed with TAM diet (40 mg/kg body weight/day, TD.130860, Harlan) from day 0 post operation until the time of sacrifice to induce Cre activity.

### Echocardiography

Eight weeks after operation, mice were lightly anesthetized by 0.8–1% isoflurane inhalation and analyzed by B-mode and M-mode echocardiography using a Vevo2100 instrument with an 18–38 MHz transducer (VisualSonics)^36^. M-mode tracings of the LV were acquired using the short-axis view, with the ultrasound beam perpendicular to the LV at the midpapillary level to determine EF, FS, wall thickness, LV inner diameter, and LV volume. LV dimensions averaged over a minimum of five consecutive cardiac cycles per heart. Doppler echocardiography was performed on all mice subjected to TAC to ensure equal pressure gradients across the aortic constriction between groups^37, 38^.

### Histology and immunofluorescence

At the time of collecting, mouse hearts were perfusion-washed with ice-cold 1× phosphate buffered saline (PBS), followed by 0.1 ml of 10% KCl to induce diastolic arrest. The hearts were then perfusion-fixed with 4% paraformaldehyde (PFA, Electron Microscopy Sciences) for 15 min. For mice not in the *R26* ^*mTmG*^ background, heart tissues were further fixed in 4% PFA overnight at 4^°^ C and subsequently processed for paraffin embedding and sectioned at 5 μm. Heart sections were deparaffinized and hydrated through a graded ethanol series (100, 95, 75, and 50%), then stained with Sirius Red to visualize fibrosis^39^ or hematoxylin and eosin^40^ to show the heart structure. For *R26* ^*mTmG*^ mice, fixed hearts were infused with 30% sucrose overnight at 4 °C and embedded in OCT (optimal cutting temperature) medium (SAKURA) prior to cryosectioning (5 µm). Heart sections from *R26* ^*mTmG*^ mice were incubated with 0.3% H_2_O_2_ in PBS at room temperature (RT) overnight in a ultraviolet chamber to quench the endogenous fluorescent signal before immunofluorescence staining^40^. The following antibodies were used for immunofluorescence: anti-collagen 1 (Col1, 1:50, Millipore, #AB765P), anti-collagen 3 (Col3, 1:50, Rockland, #600401105), anti-αSMA (1:500, Sigma, #A5228), MF20 (1:200, DSHB, #AB2147781), anti-vimentin (1:700, Abcam, #AB45939), anti-endomucin (1:250, eBioscience, #14-5851), anti-GFP (1:500, Abcam, #AB290 and #AB13970), anti-phospho-histone H3 (pHH3) (1:300; Millipore, #06-570), anti-α-actinin (1:1000, Sigma-Aldrich, #A7811), and biotinylated anti-lectin I (1:300, Vectorlabs, #B-1105). Corresponding anti-rabbit/mouse/chicken fluorescent-conjugated secondary antibodies (Molecular Probes, 1:300) were used. Alexa Fluor 488-conjugated WGA (1:300, ThermoFisher, #W11261) was used to visualize cell membranes. Nuclei were counterstained with DAPI.

Low-magnification color images were obtained using a Nikon SMZ1500 microscope, DXM1200F digital camera, and NIS-Elements BR 3.2 software. High-magnification color images were obtained using an Olympus BX51 microscope, Nikon DS-Ri1 digital camera, and NIS-Elements BR 3.2 software. Fluorescent images were captured with Zeiss LSM 510 confocal microscope and LSM version BR 3.2 software. For confocal microscopy, four to six controls and mutants were stained and visualized using Nikon confocal microscope (Nikon) with consistent settings for each group. The number of individual αSMA^+^ cells exclusive of smooth muscle cells surrounding defined blood vessels was determined by investigators blinded to genotype in microscopic fields at ×400 in the LVFW. A minimum of 10–15 fields were analyzed and averaged to obtain the number of αSMA^+^ cells per field for each sample. Similar analyses were performed for the number of vimentin^+^ and vimentin^+^;endomucin^−^ cells per field in LVFW.

### Analysis of interstitial fibrosis

To detect collagen content, rehydrated heart sections were stained in Sirius Red solution for 1 h at RT^39^. Stained sections were rinsed twice in 1% acetic acid, dehydrated through ethanol washes, cleared in xylene, and mounted with Cytoseal (Electron Microscopy Sciences). Samples were analyzed using Nikon light microscope and NIS Elements Basic Research software (Nikon) with the same light setting and pixel classifier. Interstitial fibrosis was determined as the percent of collagen-stained area/total myocardial area.

### Isolation of GFP^+^ cells from mouse hearts

Two methods were used to isolated GFP^+^ cells from heart tissue. One was through GFP antibody (Abcam, #AB290)-coated magnet beads (Dynabeads M280, Life Technologies, #11203D) pull-down^41^. Magnet beads of 2 × 10^8^ were washed three times in 0.1% BSA (bovine serum albumin) in PBS and then incubated with 5 μl anti-GFP antibody (Abcam, #AB290) at 4 °C overnight. After three washes, GFP antibody-coated magnet beads were re-suspended in 500 μl 0.1% BSA in PBS (4 × 10^8^/ml) and stored at 4 °C. Cardiac cell suspensions were prepared from collected mouse hearts perfused with cold PBS^31^. The LV was isolated, cut into small pieces, and digested in collagenase type 2 (1 mg/ml, Worthington, #LS004176)/HBSS (Hanks' balanced salt solution) (Hyclone) two times at 37 °C for 20 min. The cell suspension was filtered with 100 μm mesh into 5% fetal bovine serum (FBS, Hyclone)/HBSS. Cardiomyocytes were depleted by centrifuging at 50×*g* for 5 min. The supernatant was then transferred to another tube and centrifuged at 500×*g* for 10 min. The cell pellet was re-suspended in 0.1% BSA in PBS (4 ml for one adult heart). Magnetic beads that are 10^7^ GFP antibody-coated were incubated with 1-ml cell suspension at 4 °C for 30–60 min and then magnetically pulled down by a magnetic rack (Invitrogen). After three washes, isolated GFP^+^ cells were used for protein isolation and western blot analysis.

Alternatively, GFP^+^ cell sorting was performed using a FACSAriaII flow cytometer (BD)^42^. Cardiac cell suspensions of individual hearts were prepared as described above. Anti-red fluorescent protein antibody (Abcam, #AB62341)-coated magnet beads were used to deplete the non-recombined GFP-negative cells. The cell suspension was then sent for sorting using a FACSAriaII flow cytometer in the flow cytometry core at Cincinnati Children’s Medical Center. Sorted GFP^+^ cells were collected in Trizol reagent (Invitrogen) for mRNA analysis.

### Protein isolation and western blotting

Protein lysates were obtained from isolated GFP^+^ cells or cultured CFs. Standard western blots were performed. Briefly, 15–30 μg of protein was separated by 10–15% SDS-PAGE gel and transferred to PVDF membranes. After blocking in Odyssey blocking buffer (LI-COR, P/N 927-40000), blots were probed with primary antibodies: activated β-catenin (1:500, Millipore, #05-665), total β-catenin (1:1000, Cell signaling, #9581 S), and GAPDH (1:10 000; Santa Cruz Biotechnology #25778). After incubation with corresponding anti-mouse/rabbit secondary antibodies (1:10 000; LI-COR), immunoblots were developed using ODYSSEY Infrared Imaging System (LI-COR). Signal intensities were quantified with ImageStudio software (LI-COR). GAPDH was used as a loading control. Uncropped images of western blots are included in Supplementary Fig. 9.

### Quantitative real-time PCR

For mRNA analysis, total RNA was extracted with Trizol reagent (Invitrogen)^40^. RNA of 500 ng was used to synthesize complementary DNA using Moloney murine leukemia virus reverse transcriptase (Invitrogen, 28025-013) and random primers (Invitrogen, P/N 51709). Quantitative real-time PCR was conducted using SYBR Green PCR Master Mix (Applied Biosystems, #4367659) or Taqman probes (rat *Gapdh*, *Nppa*, and *Myh7*, Applied Biosystems). The oligonucleotide primer sequences used with SYBR green are listed in Supplementary Table 3. Samples were amplified for 35 cycles using a StepOnePlus Real-Time PCR system (Applied Biosystems). The mRNA levels normalized to L7, *Gapdh* (rat) or β-2-microglobulin (B2M) were determined using the comparative ΔΔCt method^40^.

### TUNEL staining

Heart sections from *Tcf21* ^*MCM*^ *;R26* ^*mTmG*^ *;Ctnnb1* ^*fl/fl*^ β-catenin LOF and *Tcf21* ^*MCM*^ *;R26* ^*mTmG*^ *;Ctnnb1* ^*fl/+*^ control hearts isolated 4 weeks post TAC were stained using a TUNEL kit (Roche, #11684795910) according to the manufacturer’s instructions. Briefly, sections were permeabilized with 0.1% Triton X-100 at RT for 10 min followed by incubation of TUNEL reaction mixture at 37 °C for 1 h. Sections were then co-stained with GFP antibody.

### CF cultures

CFs were cultured from post-natal day 0 (P0) and day 60 (P60) mouse hearts^31^. Cardiac cell suspensions were prepared for six to eight P0 hearts or two P60 hearts pooled for each preparation as described above for cell-sorting experiments. Cardiac cell suspensions were then plated into a 10 mm cell culture dish at 37 °C. Attached CF cells (1 h for P0 CF attachment and 4–6 h for P60) were cultured in DMEM (Corning, #15-017) supplemented with 15% FBS, 1% penicillin/streptomycin (Life Technologies), and MEM non-essential amino acid solution (ThermoFisher, #11140-050). Passage 2 CFs were used for all the in vitro experiments. Passage 2 P60 CFs were starved with serum-free DMEM overnight 24 h after seeding. The following treatments were used to manipulate CF activation and Wnt/β-catenin signaling: recombinant human Wnt1 (100 ng/ml, Invitrogen, #PHC1804), porcine TGFβ1 (10 ng/ml, R&D systems, #101-B1-001), and XAV-939 (5 μM, SelleckChem). DMSO (Sigma) was added at 0.1% as the vehicle control in DMEM.

### ChIP assay

β-catenin protein complex binding to chromosomal DNA was evaluated in mouse P0 CFs treated with 6-bromoindirubin-3′-oxime (BIO, 2 μM, Cayman Chemical) by ChIP assay^40^. DNA/protein complexes were cross-linked for 10 min in formaldehyde (Sigma, #252549) at a final concentration of 1%. The fixed cells were lysed and sonicated twice for 15 s each at output 10 (Virsonic 60; Virtis) and a 2 min refractory period. For immunoprecipitation, cell lysates were incubated with an antibody against β-catenin (5 μg; Invitrogen, #71-2700) and incubated overnight at 4 °C with gentle rocking. Immunoprecipitation with normal rabbit IgG (Upstate, #PP64B) was used as control. ChIP assays were performed according to the manufacturer’s instructions (EZChIP kit, Millipore, #17-371). The immunoprecipitations were subjected to quantitative real-time PCR with primer sets that amplify *Col1a1*, *Col3a1*, and *Postn* gene regions^43^ (murine genome GRCm38/mm10) containing potential LEF/TCF-binding sites^20^. The primer set flanking an *Axin2* intron1 region that contains previously reported LEF/TCF-binding sites was included as a positive control^20^. Primers are listed in Supplementary Table 3. Fold-enrichment relative to the IgG antibody control (negative control) set to 1.0 was calculated using the comparative CT method (ΔΔCt)^40^.

### Statistical analysis

Data were summarized using median and interquartile ranges. All data sets were analyzed using Graphpad Prism (version 6.0, La Jolla, CA). Statistical analyses between two groups were analyzed with Mann–Whitney *U* tests. For data with multiple groups, Kruskal–Wallis tests were performed, followed by Mann–Whitney *U* tests for pairwise comparisons using Bonferonni adjustments to control for multiple testing. Fold enrichment of DNA binding in ChIP assays was analyzed using one sample Wilcoxon signed-rank test compared to one. A two-tailed *p* value < 0.05 was considered to be statistically significant.

### Data availability

Data that support the reported findings are available from the corresponding author upon reasonable request.

## Electronic supplementary material

Supplementary information
