# Supplementary material for: Loss of β-catenin in resident cardiac fibroblasts attenuates fibrosis induced by pressure overload in mice
Source: Nat Commun. 2017 Sep 28;8:712. doi: 10.1038/s41467-017-00840-w (PMC5620049; doi:10.1038/s41467-017-00840-w)
Supplement: Supplementary file 1 — Supplementary information [file 41467_2017_840_MOESM1_ESM.pdf]

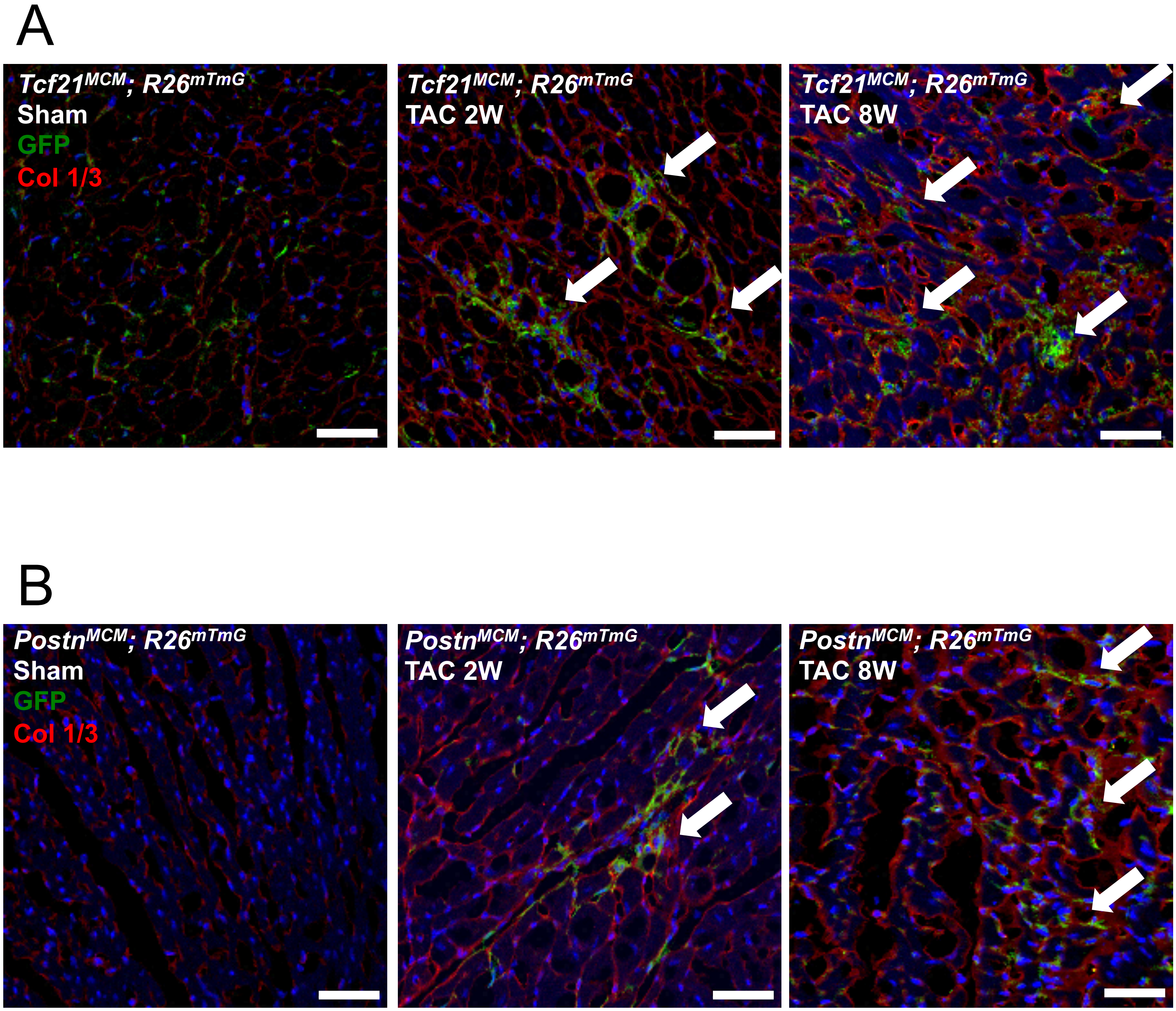

Supplementary Figure 1. Co-localization of Tcf21 and Postn lineage CFs with Collagen 1/3 in mouse hearts subjected to TAC. Representative images of GFP (Green) and Collagen 1/3 (Red) co-staining in *$Tcf21^{MCM};R26^{mTmG}$* (A) and *$Postn^{MCM};R26^{mTmG}$* (B) hearts 2 weeks (W) or 8W after sham or TAC surgeries are shown. GFP^{+} cells are present in areas of Col1/3 deposition (arrows). Scale bar=50µm.

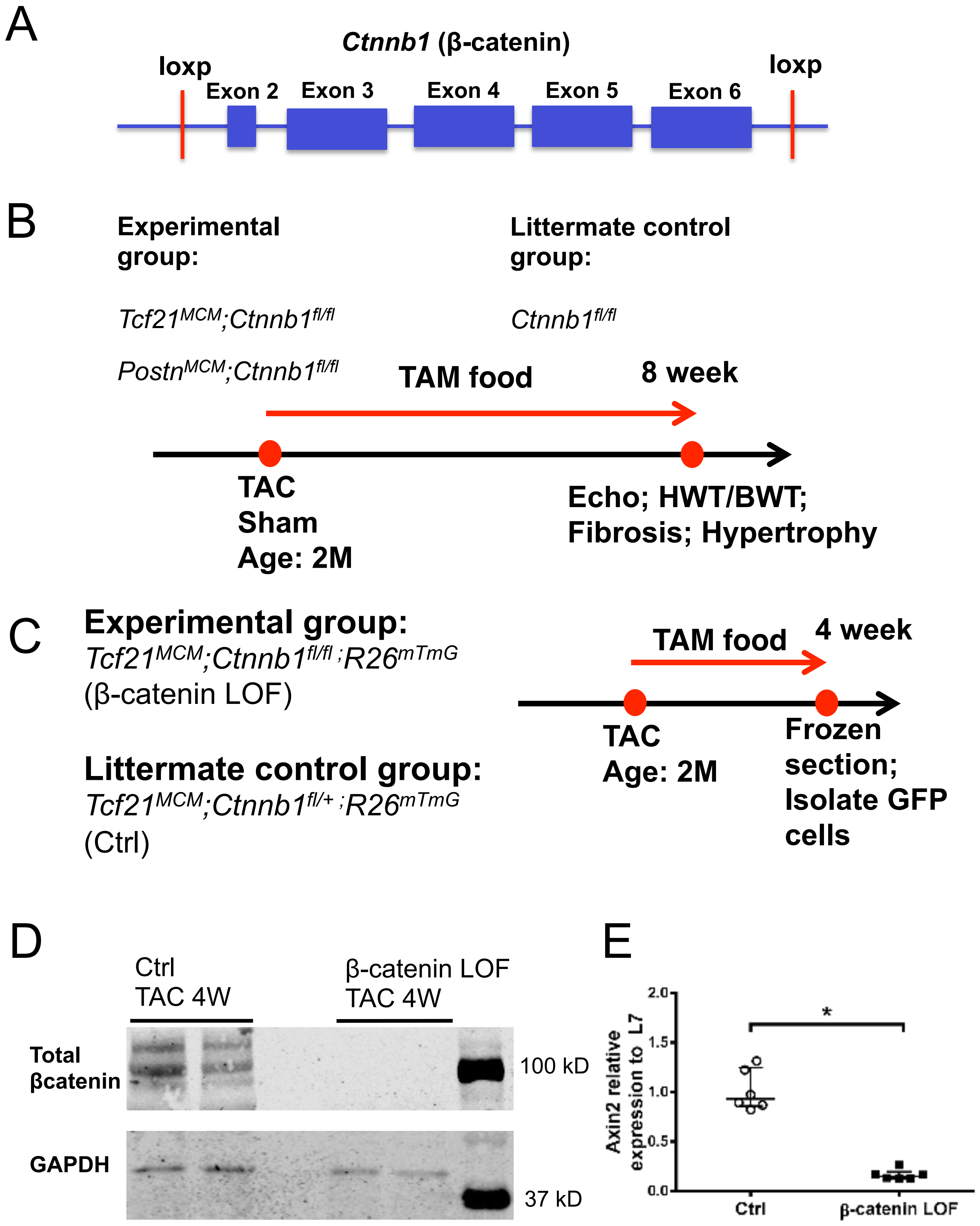

A
Ctnnb1 (β-catenin)
loxp
Exon 2
Exon 3
Exon 4
Exon 5
Exon 6
loxp
B
Experimental group:
Tcf21MCM;Ctnnb1fl/fl
PostnMCM;Ctnnb1fl/fl
Littermate control group:
Ctnnb1fl/fl
TAM food
8 week
TAC
Sham
Age: 2M
Echo; HWT/BWT;
Fibrosis; Hypertrophy
C
Experimental group:
Tcf21MCM;Ctnnb1fl/fl;R26mTmG
(β-catenin LOF)
Littermate control group:
Tcf21MCM;Ctnnb1fl/+;R26mTmG
(Ctrl)
TAM food
4 week
TAC
Age: 2M
Frozen section;
Isolate GFP cells
D
Ctrl
TAC 4W
β-catenin LOF
TAC 4W
Total βcatenin
GAPDH
100 kD
37 kD
E
Axin2 relative expression to L7
2.0
1.5
1.0
0.5
0.0
*
Ctrl
β-catenin LOF

Supplementary Figure 2. Experimental design for conditional β-catenin loss-of-function (LOF) in adult Tcf21 and Postn lineages after cardiac pressure overload (TAC). A) Illustration of the β-catenin LOF locus with *loxp* sites flanking Exons 2 and 6 of the *Ctnnb1* gene. B) $Tcf21^{MCM};Ctnnb1^{fl/fl}$ and $Postn^{MCM};Ctnnb1^{fl/fl}$ mice were used for CF-specific β-catenin LOF. Cre-negative $Ctnnb1^{fl/fl}$ littermate mice were used as control groups. At the age of 2 months, both groups were randomly subjected to either sham or TAC surgery. CF-specific β-catenin LOF was induced after surgery via tamoxifen food (≈40 mg/body weight/day). Mice were maintained on tamoxifen food until termination. Eight weeks post-surgery, echocardiography was performed and mice were sacrificed. Heart samples were harvested for further experiments. C) $Tcf21^{MCM};R26^{mTmG};Ctnnb1^{fl/fl}$ (β-catenin LOF) mice were used to label and trace Tcf21 lineage CFs with β-catenin LOF. $Tcf21^{MCM};R26^{mTmG}$; $Ctnnb1^{fl/+}$ (control) mice were used as controls. At 2 months, both groups were randomly subjected to TAC or sham surgery. CF-specific β-catenin LOF was induced right after surgery via tamoxifen food (≈40 mg/body weight/day). Mice were maintained on tamoxifen food for 4 weeks. Four weeks post-surgery, hearts were collected for immunostaining or isolation of GFP-labeled CFs. D) Loss of β-catenin in GFP^+ cells from $Tcf21^{MCM};R26^{mTmG};Ctnnb1^{fl/fl}$ (β-catenin LOF) hearts was confirmed by Western blot analysis in N=2 experiments of 3 hearts each. E) Expression of *Axin2* mRNA was also significantly reduced in GFP^+ cells from $Tcf21^{MCM};R26^{mTmG};Ctnnb1^{fl/}$ β-catenin LOF hearts compared to controls. N=6 hearts/group. Data points are shown with median and interquartile ranges indicated. Statistical significance was determined using unpaired Mann-Whitney U tests: $^*P<0.05$ versus control.

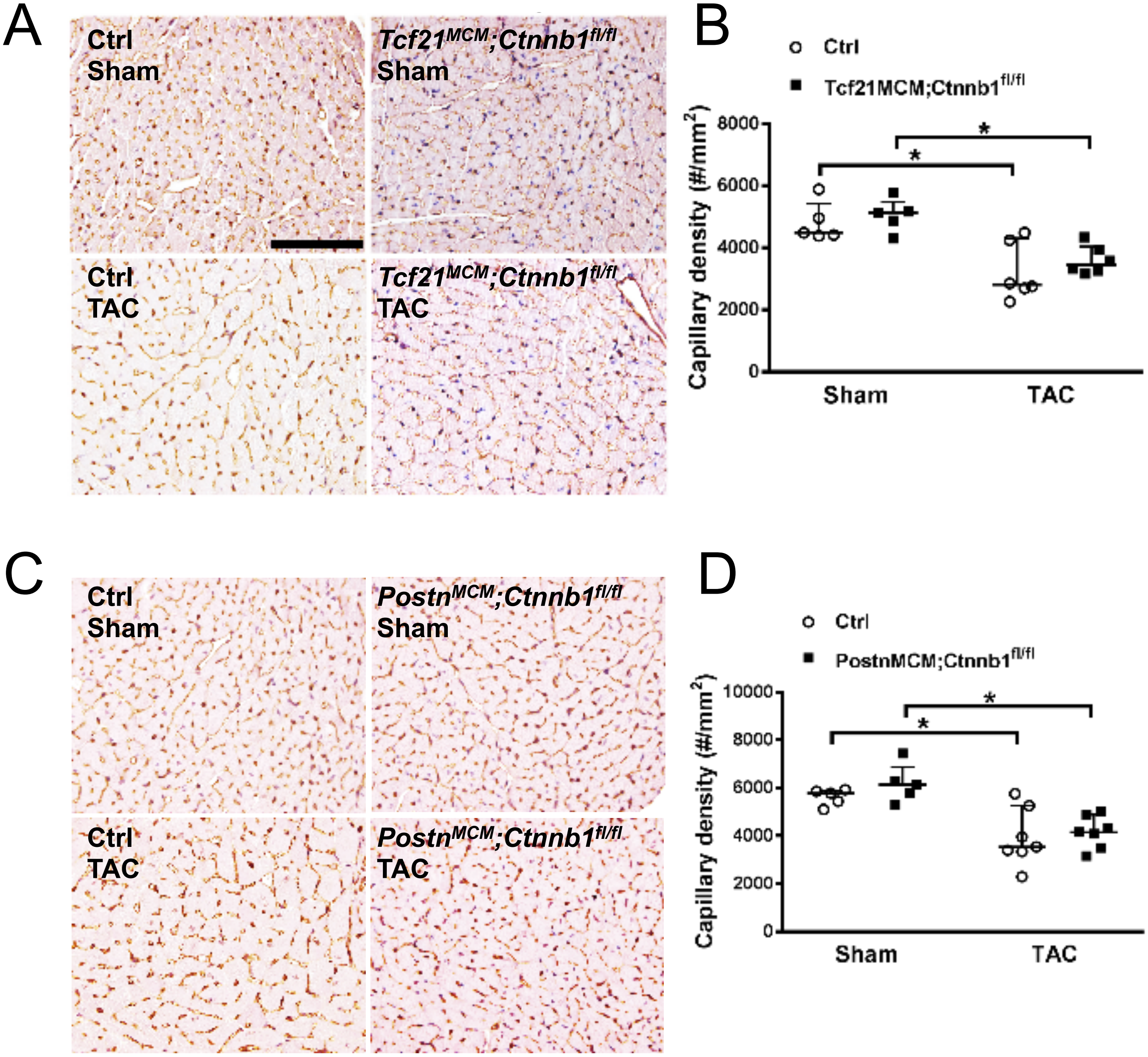

Supplementary Figure 3. Capillary density 8 weeks after TAC. A,C) Representative images of Lectin1 staining in Ctrl and β-catenin LOF hearts 8 weeks after TAC are shown. Myocardial capillary density significantly decreased 8 weeks after TAC but no difference was observed between Ctrl and *Tcf21*^{MCM};*Ctnnb1*$^{fl/fl}$ (B) and *Postn*^{MCM};*Ctnnb1*$^{fl/fl}$ mice (D) N=5-7 hearts/group. Data points are shown with median and interquartile ranges indicated. Statistical significance was determined using unpaired Mann-Whitney U tests: *$P<0.05$ vs Ctrl. Scale bar=100μm.

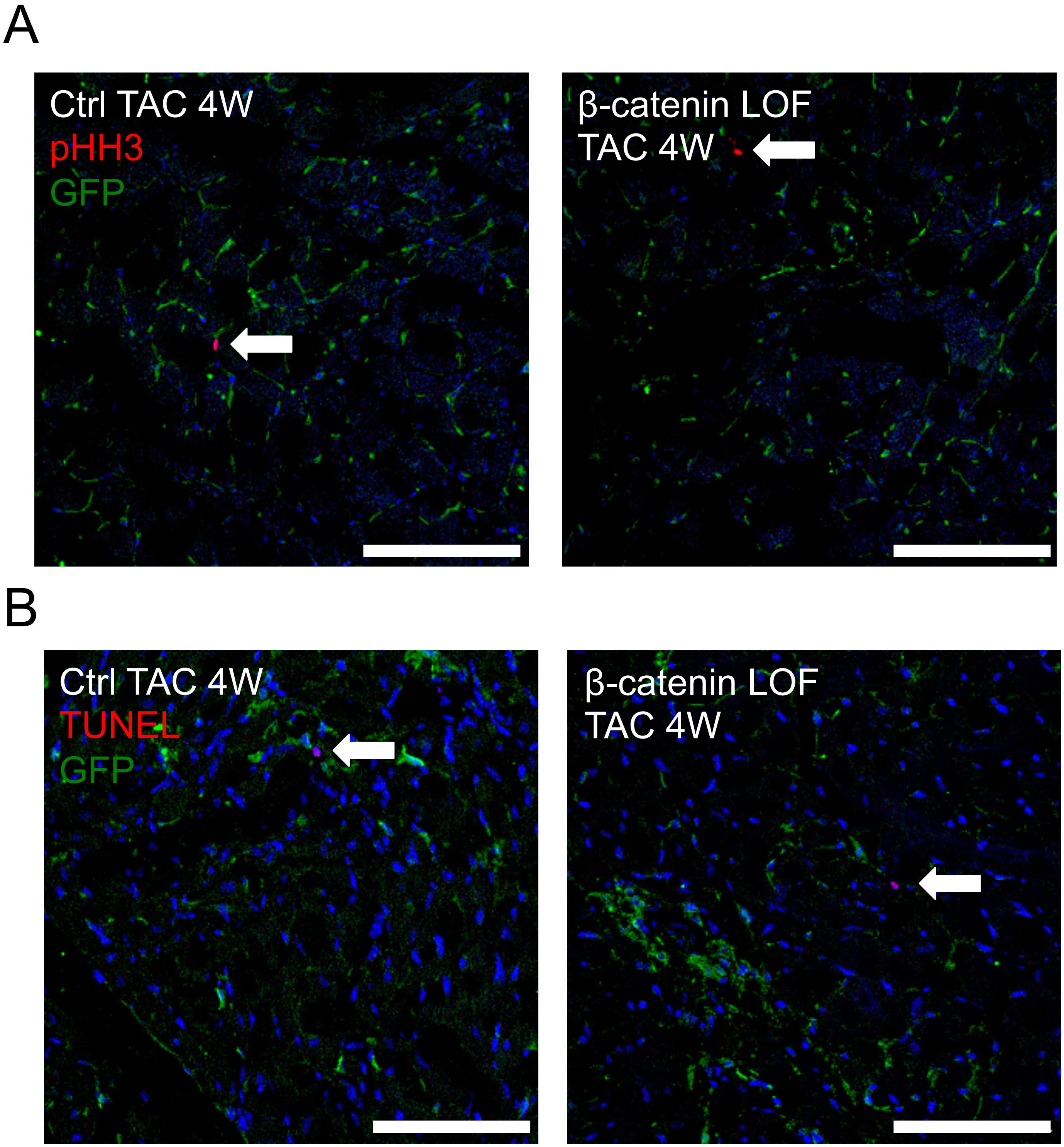

Supplementary Figure 4. Proliferation and apoptosis were barely detected in GFP^+ Tcf21 lineage CFs 4 weeks after TAC. A) Representative images of GFP (Green) and pHH3 (Red) co-staining in *Tcf21*^{MCM} β-catenin LOF and control hearts 4 weeks after TAC are shown. B) Representative images of GFP (Green) and TUNEL (Red) co-staining in β-catenin LOF and control hearts 4 weeks after TAC are shown. Very few $pHH3^+/GFP^+$ and $TUNEL^+/GFP^+$ cells (white arrows) were found in myocardium. Scale bar=100μm.

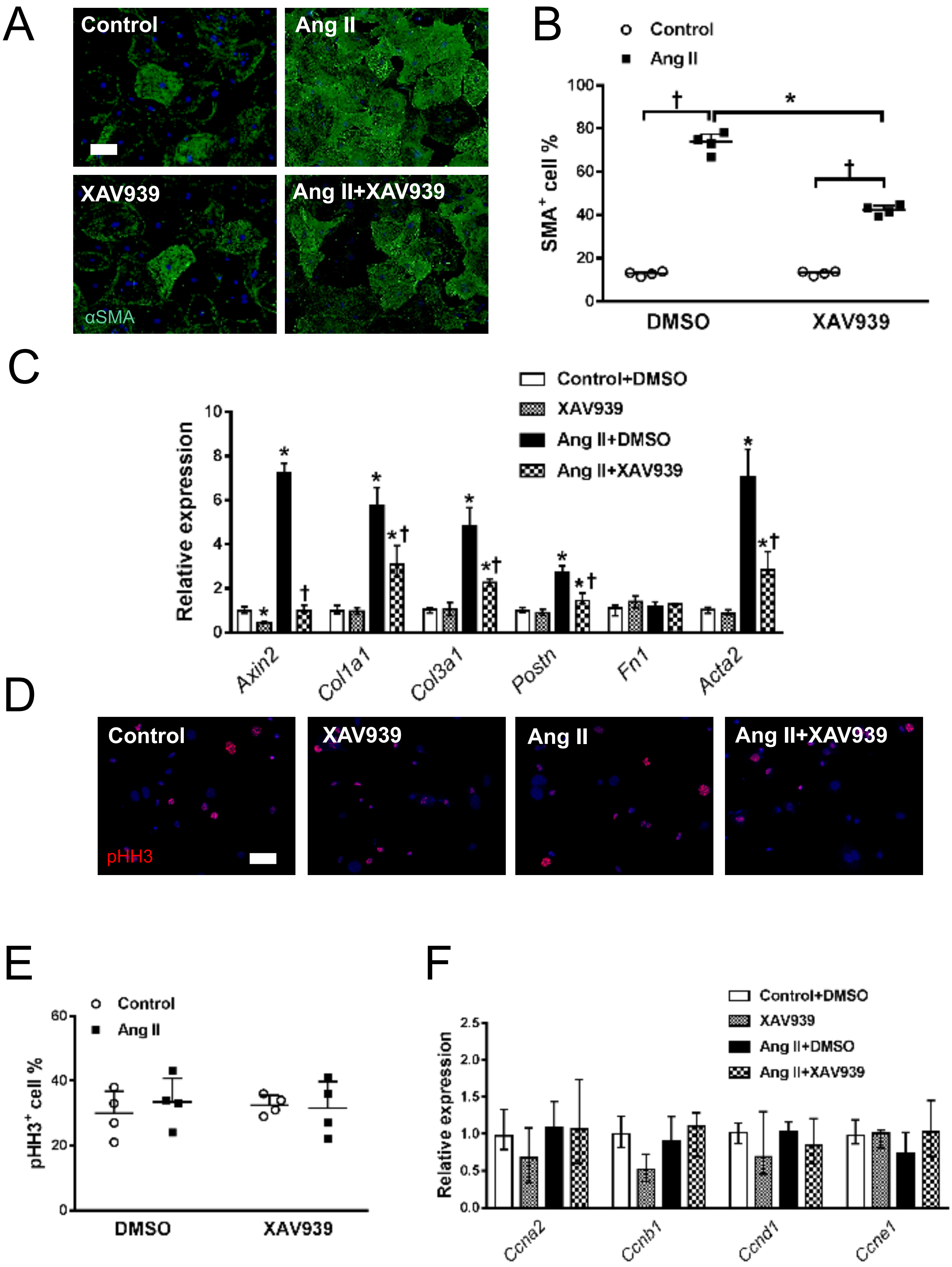
A
Control
Ang II
XAV939
Ang II+XAV939
αSMA
B
Control
Ang II
SMA+ cell %
DMSO
XAV939
C
Control+DMSO
XAV939
Ang II+DMSO
Ang II+XAV939
Relative expression
Axin2
Col1a1
Col3a1
Postn
Fn1
Acta2
D
Control
XAV939
Ang II
Ang II+XAV939
pHH3
E
Control
Ang II
pHH3+ cell %
DMSO
XAV939
F
Control+DMSO
XAV939
Ang II+DMSO
Ang II+XAV939
Relative expression
Ccna2
Ccnb1
Ccnd1
Ccne1

Supplementary Figure 5. Wnt/β-catenin signaling contributes to Angiotensin II (Ang II) induced cardiomyocyte CF activation. A) Representative images of αSMA (Green) staining in cultured P60 CFs under indicated conditions are shown. Scale bar=100μm. B) Ang II (100nM) induces significantly more αSMA+ CFs relative to DMSO controls in cultured P60 CFs. Inhibition of Wnt/β-catenin signaling by XAV939 (5μM) partially abrogated Ang II-induced αSMA expression in CF. *P<0.05 vs DMSO groups, † P<0.05 vs. Control . C) mRNA expression of the Wnt target gene *Axin2*, and ECM genes *Col1a1*, *Col3a1*, *Postn* and *Acta2* in P60 CFs with indicated treatments was measured by qPCR. *P<0.05 vs control,† P<0.05 vs. DMSO. D) Representative images of pHH3 (Red) staining in cultured P60 CFs under indicated conditions are shown. Scale bar=100μm. No difference in proliferating CF percentage (E) and cell-cycle genes (F) *Ccna2*, *Ccnb1*, *Ccnd1* and *Ccne1* expression was observed among groups. N=4 independent cultures/group. Statistical significance was determined by Kruskal-Wallis tests followed Mann-Whitney U tests for pairwise comparisons using Bonferonni adjustments to control for multiple testing. Data points are shown with median and interquartile ranges indicated.

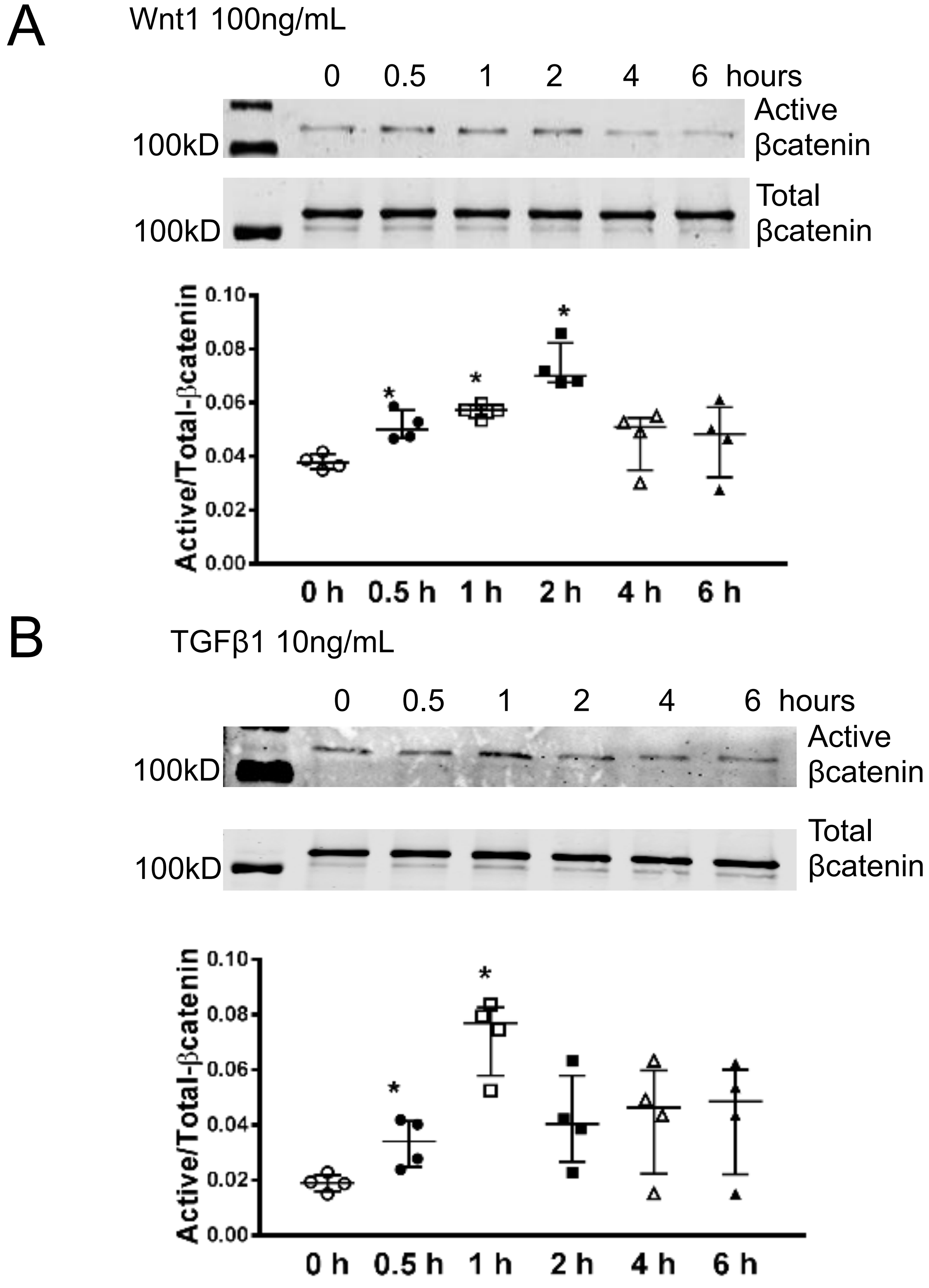

Supplementary Figure 6. Wnt1 and TGFβ1 induce β-catenin activation in cultured P60 CFs. A) Wnt1 induces β-catenin activation in cultured P60 CFs as determined by Western analysis. B) TGFβ1 induces β-catenin activation in cultured P60 CFs as determined by Western analysis. Data points are shown with median and interquartile ranges indicated. Statistical significance was determined using unpaired Mann-Whitney U tests: *$P<0.05$ versus 0 h. N=4 independent cultures.

A

ChIP sequence tracks for ***Col3a1***

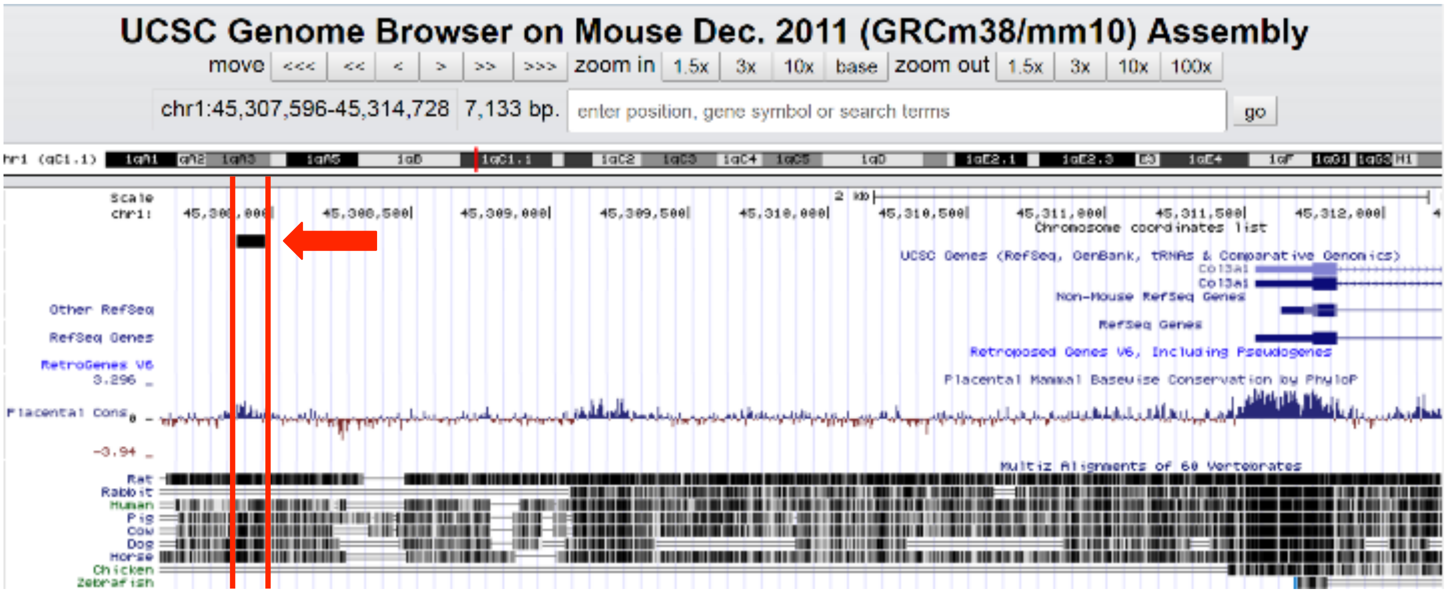

B

ChIP sequence tracks for ***Postn***

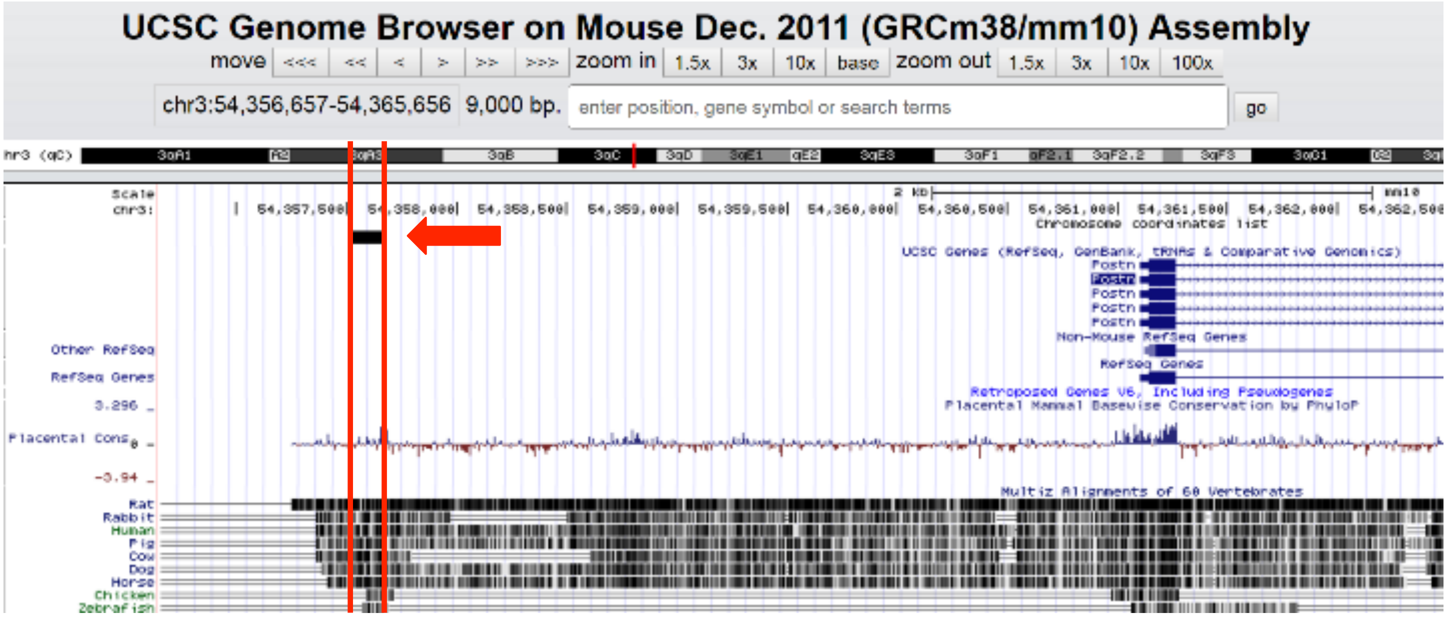

Supplementary Figure 7. Genomic sequences containing LEF binding domains for *Col3a1* and *Postn* loci amplified in β-catenin ChIP assays. Screen shots of UCSC genome browser21 (UCSC Genome Browser on Mouse Dec 2011 (GRCm38/mm10)) show LEF binding domains (red arrow) for Col3a1 (A) and Postn (B) that were amplified in β-catenin ChIP assays. These sequences have high conservation among species.

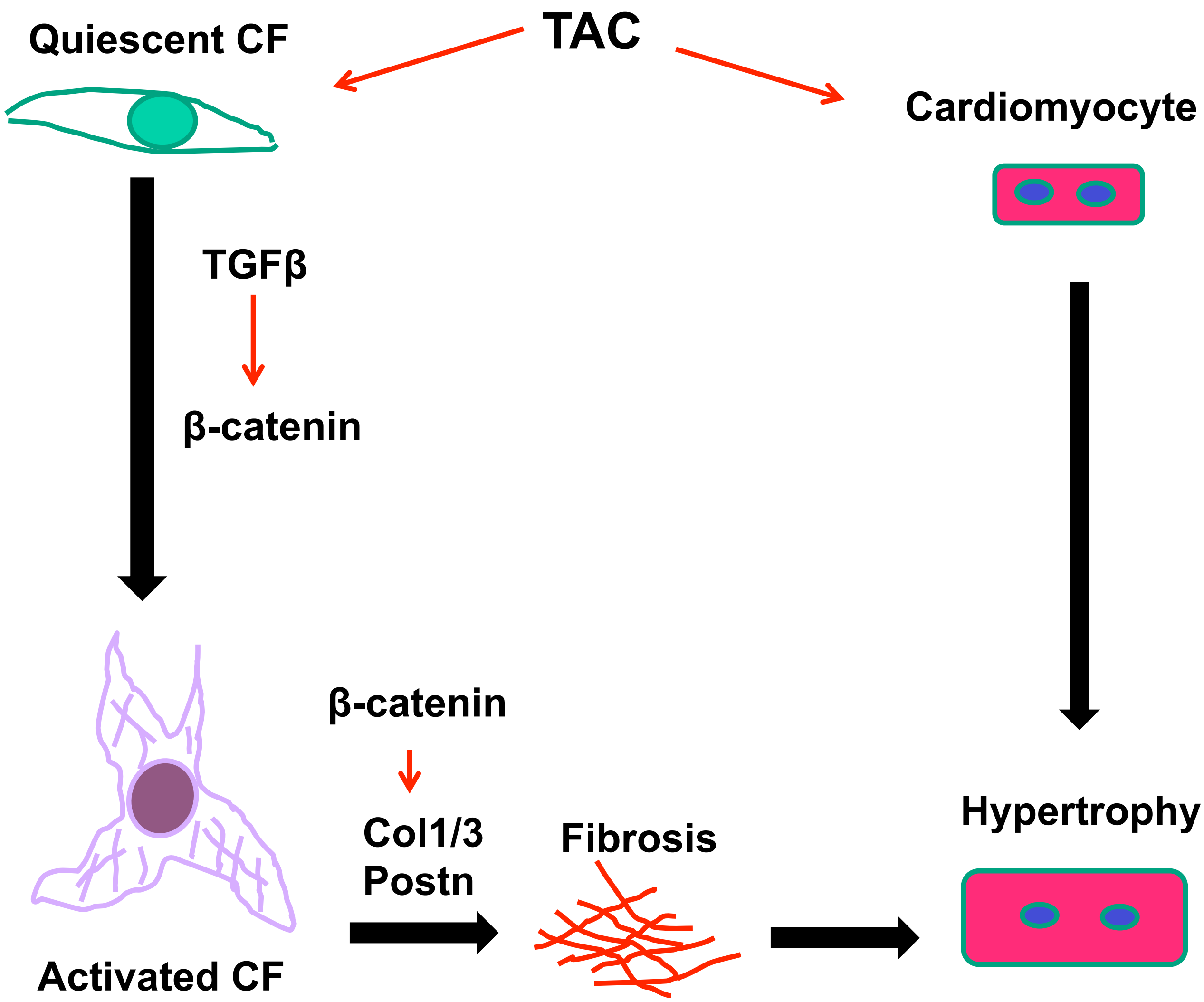

Supplementary Figure 8. Model for Wnt/β-catenin signaling in CFs after pressure-overload injury induced by TAC. In TAC-induced pressure overload injury, Wnt/β-catenin is required for the synthetic phenotype in CFs as indicated by fibrotic gene expression, including *Col1a1*, *Col3a1* and *Postn*. With loss of β-catenin in Tcf21or Postn lineage cells, reduced fibrotic ECM gene expression and cardiomyocyte hypertrophy is the primary outcome after TAC-induced pressure overload injury in mice. Moreover, Wnt/β-catenin may also act downstream in TGFβ induced CF activation. Therefore, Wnt/β-catenin in CFs plays an important and integral role for fibrosis and hypertrophy development.

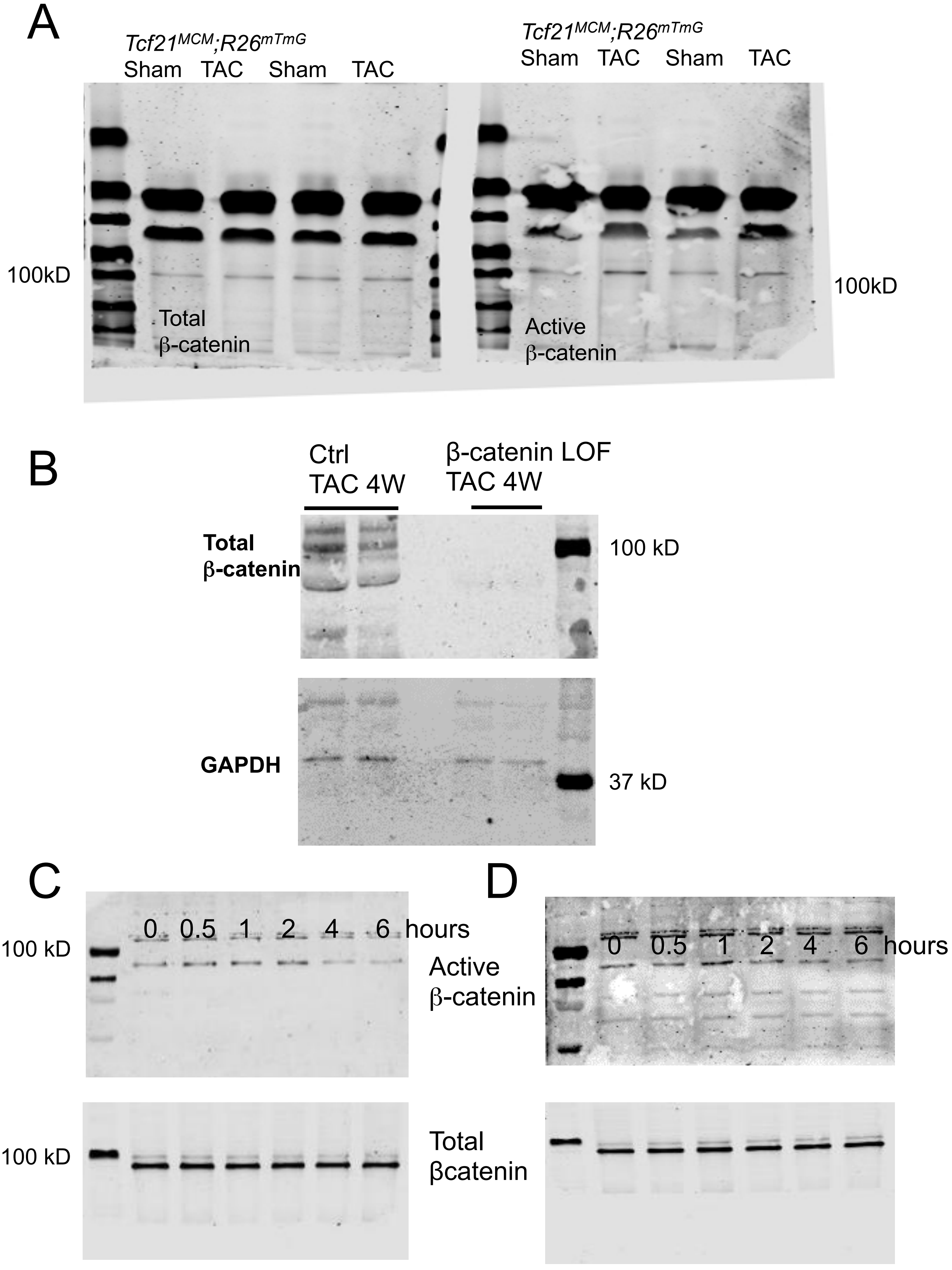

A
Tcf21MCM;R26mTmG
Sham TAC Sham TAC
100kD
Total β-catenin
Tcf21MCM;R26mTmG
Sham TAC Sham TAC
100kD
Active β-catenin
B
Ctrl TAC 4W
β-catenin LOF TAC 4W
Total β-catenin
100 kD
GAPDH
37 kD
C
100 kD
0 0.5 1 2 4 6 hours
Active β-catenin
100 kD
Total βcatenin
D
0 0.5 1 2 4 6 hours

Supplementary Figure 9. Original Western blot images. Original Western blot images are shown for Figure 1C (A), Supplementary Figure 2D (B), Supplementary Figure 6A (C), and Supplementary Figure 6B (D).

Supplementary Table 1: Cardiac function measured by echocardiography 8 weeks post-TAC or sham surgery:

	**Control sham N=5**	***Tcf21^{MCM};Ctnnb1$^{fl/fl}$*** **sham N=5**	**Control MI N=6**	***Tcf21^{MCM};Ctnnb1$^{fl/fl}$*** **MI N=6**
**IVSd (mm)**	0.78±0.03	0.72±0.09	1.07±0.07*	1.09±0.07*
**IVSs (mm)**	1.17±0.05	1.04±0.12	1.26±0.05	1.37±0.08
**LVIDd (mm)**	3.67±0.16	3.87±0.06	5.20±0.15*	4.22±0.08*†
**LVIDs (mm)**	2.51±0.13	2.59±0.11	4.55±0.17*	3.38±0.10*†
**LVPWd (mm)**	0.89±0.09	0.70±0.12	1.05±0.07	1.03±0.03
**LVPWs (mm)**	1.10±0.10	1.00±0.13	1.15±0.07	1.17±0.03
**EF (%)**	60.61±1.88	62.33±2.84	26.98±2.35*	41.16±2.01*†
**FS (%)**	31.78±1.30	33.24±1.97	12.66±1.19*	19.95±1.14*†
**LV mass**	109.67±15.40	96.18±17.04	262.83±17.41*	187.21±11.35*†
**LV mass corrected**	88.34±12.32	77.55±13.63	210.86±13.93*	150.36±9.08*†
**LV Vol d (µL)**	57.89±6.21	64.99±2.46	130.40±8.38*	79.54±3.59*†
**LV Vol s (µL)**	22.90±2.79	24.72±2.72	95.86±8.49*	47.01±3.34*†
**HR (bpm)**	448.80±11.28	458.20±12.94	441.50±5.96	443.67±10.51
**Pressure gradient (mmHg)**			51.17±1.72	50.50±2.17

	**Control sham N=5**	***Postn^{MCM};Ctnnb1$^{fl/fl}$*** **sham N=5**	**Control MI N=6**	***Postn^{MCM};Ctnnb1$^{fl/fl}$*** **MI N=6**
**IVSd (mm)**	0.95±0.05	0.90±0.03	1.12±0.05	1.08±0.08
**IVSs (mm)**	1.21±0.16	1.19±0.08	1.30±0.04	1.37±0.09
**LVIDd (mm)**	4.02±0.15	4.18±0.06	5.16±0.06*	4.33±0.14*†
**LVIDs (mm)**	2.83±0.15	2.91±0.07	4.75±0.07*	3.50±0.15*†
**LVPWd (mm)**	0.62±0.04	0.65±0.01	1.20±0.12*	1.05±0.10
**LVPWs (mm)**	1.01±0.08	1.11±0.02	1.25±0.11	1.23±0.12
**EF (%)**	57.44±2.48	58.31±1.87	17.27±1.62*	39.90±2.47*†
**FS (%)**	29.88±1.65	30.52±1.27	7.85±0.76*	19.32±1.35*†
**LV mass**	114.58±8.92	119.11±2.61	293.46±17.26*	196.82±18.56*†
**LV mass corrected**	92.27±7.13	95.89±2.09	235.37±13.81*	158.06±14.85*†
**LV Vol d (µL)**	71.57±6.47	77.80±2.49	127.19±3.23*	85.26±6.44*†
**LV Vol s (µL)**	30.82±4.12	32.49±1.98	105.32±3.86*	51.82±5.28*†
**HR (bpm)**	448.60±15.3	457.20±15.86	453.86±8.21	454.57±11.77
**Pressure gradient (mmHg)**			52.57±2.89	52.43±2.89

Data are mean ± SEM. Significance was determined using Kruskal-Wallis test followed by Bonferroni corrections. *$P<0.05$ vs. sham; † $P<0.05$ vs. STG MI. IVSd: interventricular septum thickness in diastole; IVSs: interventricular septum thickness in systole; LVIDd: left ventricular end-diastolic diameter; LVIDs: left ventricular end-systolic diameter; LVPWd: left ventricle posterior wall thickness in diastole/systole; LVPWs: left ventricle posterior wall thickness in diastole/systole; EF: ejection fraction; FS: fractional shortening; LV Vol d: left ventricular end-diastolic volume; LV Vol s: left ventricular end-systolic volume; HR: heart rate.

Supplementary Table 2: Cardiac function measured by echocardiography after tamoxifen treatment for 8 weeks:

	Tcf21$^{MCM+/-}$<br><br>N=6	***Tcf21***$^{MCM-/-}$ ***Ctnnb1***$^{fl/fl}$<br>N=6	***Postn***$^{MCM+/-}$<br><br>N=6	***Postn***$^{MCM-/-}$ ***Ctnnb1***$^{fl/fl}$<br>N=6
**IVSd (mm)**	0.78±0.02	0.78±0.03	0.93±0.02	0.91±0.02
**IVSs (mm)**	1.15±0.02	1.27±0.07	1.30±0.03	1.42±0.09
**LVIDd (mm)**	3.53±0.14	3.79±0.07	3.68±0.13	3.94±0.07
**LVIDs (mm)**	2.35±0.12	2.48±0.08	2.50±0.12	2.66±0.08
**LVPWd (mm)**	0.79±0.07	0.74±0.04	0.94±0.07	0.89±0.05
**LVPWs (mm)**	1.00±0.08	1.07±0.08	1.15±0.08	1.22±0.08
**EF (%)**	63.35±2.07	64.66±1.65	61.26±2.11	61.64±1.37
**FS (%)**	33.67±1.48	34.79±1.19	32.28±1.45	32.67±0.94
**LV mass**	93.67±11.51	99.63±3.60	126.76±11.01	134.23±5.58
**LV mass corrected**	75.53±9.21	80.30±2.88	102.01±8.81	107.99±4.46
**LV Vol d (µL)**	52.60±5.15	61.89±2.70	57.96±4.96	67.9±3.02
**LV Vol s (µL)**	19.48±2.48	22.00±1.82	22.64±2.53	26.21±1.99
**HR (bpm)**	454.33±9.52	453.17±8.74	453.17±9.46	450.17±8.70

Data are mean ± SEM.

Supplementary Table 3: Primers for qRT-PCR and ChIP

**Genotyping primers**		
**Gene**	**Forward Sequence**	**Reverse Sequence**
Cre	GCGGTCTGGCAGTAAAAACTATC	GTGAAACAGCATTGCTGTCACTT
Ctnnb1$^{fl/fl}$	AAGGTAGAGTGATGAAAGTTGTT	CACCATGTCCTCTGTCTATTC
ROSA^{mTmG}	CTCTGCTGCCTCCTGGCTTCT	TCAATGGGCGGGGGTCGTT
**qRT-PCR primers**		
**Gene**	**Forward Sequence**	**Reverse Sequence**
L7	AAGACGAAGGAGCTGCAGAAC	GAAGCTCATCTATGAGAAGGC
Axin2	GAGTAGCGCCGTGTTAGTGACT	CCAGGAAAGTCCGGAAGAGGTATG
Col1a1	TCCTGACGCATGGCCAAGAAGACA	TCCGGGCAGAAAGCACAGCACTC
Col3a1	GCACAGCAGTCCACCGTAGA	TCTCCAAATGGGATCTCTGG
Postn	CGAAGGGGACAGTATCTCCA	AGGTCGGTGAAAGTGGTTTG
Fn1	ATGTGGACCCCTCCTGATAGT	GCCCAGTGATTTCAGCAAAGG
Acta2	GTCCCAGACATCAGGGAGTAA	TCGGATACTTCAGCGTCAGGA
Myh7	ATGTGCCGGACCTTGGAAG	CCTCGGGTTAGCTGAGAGATCA
**ChIP primers**		
**Gene**	**Forward Sequence**	**Reverse Sequence**
Axin2	P+1828: TCCCGTGTCACTGTTTCT	P+1944: AGGTGCTCGTCTCAAGTAT
Col3a1	P-4700: ACCAACAGATTGGGAAAGG	P-4566: CTCGGGTGAGAATTCTTTGT
Postn	P-3585: GGAAGAGACTGCTAATTCCTAC	P-3458: GAGACATCTAGTGGAGAAAGTG
negative	ATGGTTGCCACTGGGGATCT	TGCCAAAGCCTAGGGGAAGA
